# Catalyzed M–C coupling reactions in the synthesis of σ-(pyridylethynyl)dicarbonylcyclopentadienyliron complexes†

**DOI:** 10.1039/d0ra02333g

**Published:** 2020-04-30

**Authors:** Victor V. Verpekin, Oleg V. Semeikin, Alexander D. Vasiliev, Alexander A. Kondrasenko, Yuri A. Belousov, Nikolai A. Ustynyuk

**Affiliations:** Institute of Chemistry and Chemical Technology SB RAS, Krasnoyarsk Research Center, Siberian Branch of the Russian Academy of Sciences Akademgorodok 50-24 Krasnoyarsk 660036 Russian Federation vvv@sany-ok.ru; A. N. Nesmeyanov Institute of Organoelement Compounds, Russian Academy of Sciences ul. Vavilova 28 Moscow 119991 Russian Federation ustynyuk@ineos.ac.ru; Institute of Physics SB RAS, Krasnoyarsk Research Center, Siberian Branch of the Russian Academy of Sciences Akademgorodok, 50-38 Krasnoyarsk 660036 Russia; Siberian Federal University Svobodny Prospect, 79 Krasnoyarsk 660041 Russia

## Abstract

The reactions between terminal ethynylpyridines, (trimethylsilyl)ethynylpyridines and cyclopentadienyliron dicarbonyl iodide were studied under Pd/Cu-catalyzed conditions to develop a synthetic approach to the σ-alkynyl iron complexes Cp(CO)_2_Fe–C≡C–R (R = *ortho*-, *meta*-, *para*-pyridyl). Depending on the catalyst and reagents used, the yields of the desired σ-pyridylethynyl complexes varied from 40 to 95%. In some cases the reactions with *ortho*-ethynylpyridine gave as byproduct the unexpected binuclear FePd μ-pyridylvinylidene complex [Cp(CO)Fe{μ_2_-η^1^(C_α_):η^1^(C_α_)-κ^1^(N)-C_α_=C_β_(H)(*o*-C_5_H_4_N)}(μ-CO)PdI]. The conditions, catalysts, and reagents that provide the highest yields of the desired σ-pyridylethynyl iron compounds were determined. The methods developed allowed the synthesis of the corresponding σ-4-benzothiadiazolylethynyl complex Cp(CO)_2_Fe–C≡C–(4-C_6_H_3_N_2_S) as well. Eventually, synthetic approaches to σ-alkynyl iron complexes of the type Cp(CO)_2_Fe–C≡C–R (R = *ortho*-, *meta*-, *para*-pyridyl, 4-benzothiadiazol-2,1,3-yl) based on the Pd/Cu-catalyzed cross-coupling reactions were elaborated.

## Abstract 2

Two approaches were developed for the synthesis of iron σ-pyridylethynyl complexes based on Pd/Cu- and Pd-catalyzed Fe–C coupling reactions.

## Graphical abstract

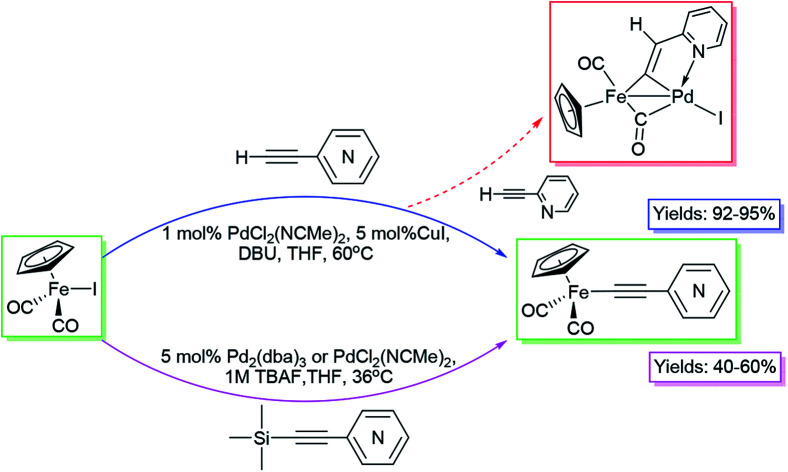

## Introduction

Metal σ-alkynyl complexes displaying such peculiar characteristics as linear geometry, high stability, and π-unsaturated character have been demonstrated to constitute promising building blocks for the design of materials, which can possess such properties as optical nonlinearity,^1–5^ light-emission,^6–12^ and electrical conductivity.^13–18^ Moreover, they are an important class of coordination compounds because of their relevance in synthetic chemistry^19–24^ and proton reduction catalysis.^25–27^

A variety of methods for the synthesis of transition metal acetylides have been developed.^24^ The most common synthetic route to them is transmetallation, where a generated [M–C≡C–R] species [M = Cu(i), Ag(i), Au(i), alkali-metal (Li, Na), or an alkaline-earth-metal (MgX, *etc.*)] acts as an alkynyl transfer reagent to transition-metal halide complexes L_*n*_MX (*X* = I, Br, Cl).^28–34^ However, in some cases, such reactions may give low yields of desired products and a range of by-products, for example, complexes in which the copper or the silver fragments are π-coordinated to the transferred alkynyl ligand.^35–38^

Other general strategy to the preparation of transition metal acetylides takes advantage of the facility of some transition metal complexes to catalyze M–C coupling reactions. The most general route to group 10 metal σ-alkynyl derivatives is based on copper(i)-catalyzed dehydrogalogenation reactions between an appropriate metal halide complex and a terminal alkyne in an amine solvent.^39–42^ This method is applicable to the synthesis of alkynyl, polyynyls, and polyyndiyls of tungsten, molybdenum, iron, ruthenium, rhodium, and iridium.^5,43–47^ In some cases, the CuI-catalyzed reactions of transition metal halides with stannyl acetylenes may be performed in the absence of amines.^48–51^

Palladium catalysts, currently being an indispensable tool of organic synthesis, can also be used in the M–C≡C– bond formation. The team of Claudio Lo Sterzo demonstrated that, similarly to organic electrophiles, transition metal iodides undergo coupling with trialkyltin acetylides, in the presence of palladium to form alkynyl complexes of ruthenium, iron, tungsten, and molybdenum.^52–55^ The role of palladium catalysts in promoting these transformations was also investigated.^52,56–59^

Although, the Lo Sterzo approach was shown to be a valuable route to alkynyl complexes, the need for preparation of tin reagents and removal of tin impurities limits the appeal of this method. To overcome these disadvantages, one can use an organometallic analogue of the Sonogashira protocol,^60,61^ where transition metal halides react with terminal alkynes to form metal alkynyls *via* the Pd/Cu- or Pd-catalyzed dehydrohalogenation route, which provides milder reaction conditions and facilitates purification of products. Despite the seeming availability of this approach for the synthesis of metal acetylides, it was exploited only once by Oshima^62^ in the synthesis of σ-alkynyl iron complexes Cp(CO)_2_Fe–C≡C–Ar.

**Scheme 1:**
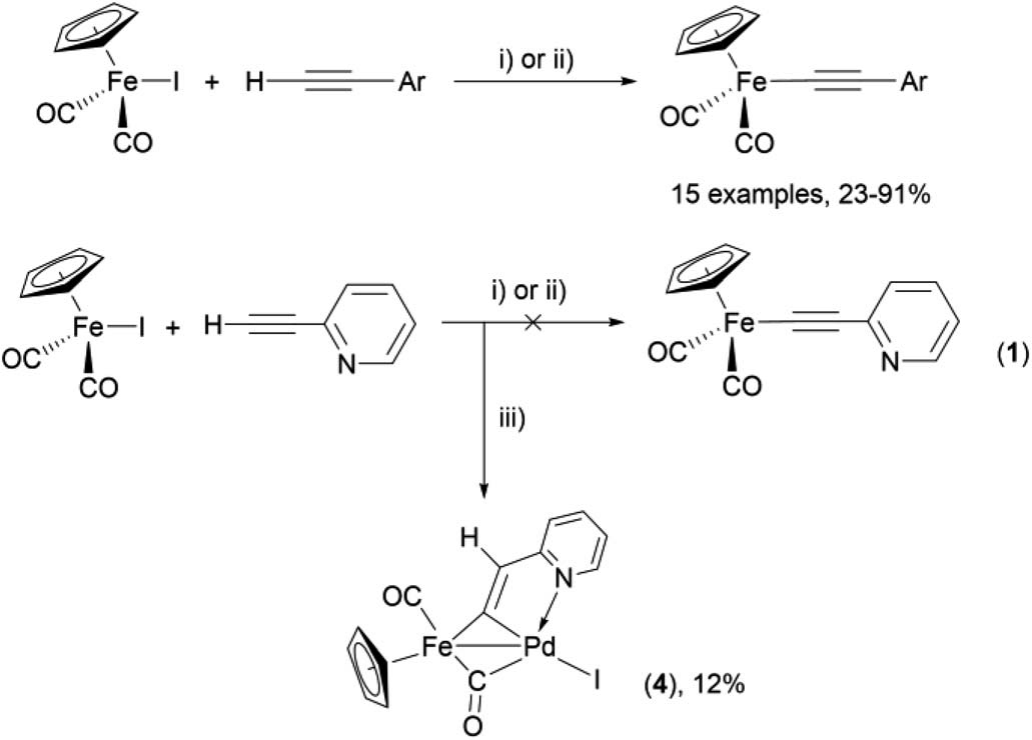
Reactions of terminal arylacetylenes^62^ and 2-ethynylpyridine with Cp(CO)_2_FeI under Oshima conditions; unexpected formation of binuclear μ-pyridylvinylidene complex 4. (i) PdCl_2_(PPh_3_)_2_ 2.5 mol%, CuI 5 mol%, ^i^Pr_2_NH–THF 1 : 2, 25 °C, 30 min; (ii) PdCl_2_(PPh_3_)_2_ 5 mol%, CuI 2.5 mol%, ^i^Pr_2_NH–THF 1 : 2, 25 °C, 30 min; (iii) 1 equiv. PdCl_2_, ^i^Pr_2_NH, 60 °C, 1 h.

As a part of our studies on proton reduction catalysis,^25^ we searched for a facile synthetic route to a series of σ-pyridylethynyl iron complexes Cp(CO)_2_Fe–C≡C–(n-C_5_H_4_N) (n = *ortho* (1), *meta* (2), *para* (3)) containing two Lewis base centers (C_β_ of ethynyl and N atom of pyridine). The efficient preparation of the iron arylethynyls complexes Cp(CO)_2_Fe–C≡C–Ar by Pd/Cu-catalyzed cross-coupling of Cp(CO)_2_FeI with terminal arylacetylenes^62^ inspired us to apply the Oshima protocol for the preparation of 1–3. Unfortunately, our first attempt to obtain the *ortho*-pyridylethynyl iron complex Cp(CO)_2_Fe–C≡C–(2-C_5_H_4_N) (1) using the Oshima conditions did not give the target substance. Upon an increase in the reaction temperature to 60 °C an unexpected binuclear FePd μ-pyridylvinylidene complex [Cp(CO)Fe{μ_2_-η^1^(C_α_):η^1^(C_α_)-κ^1^(N)-C_α_=C_β_(H)(2-C_5_H_4_N)}(μ-CO)PdI] (4) was obtained in yield of 2%, but still without traces of 1 (Scheme 1). The 12% yield of complex 4 was achieved by using one equivalent of PdCl_2_ without copper iodide and pure diisopropylamine as a solvent^63^ (Scheme 1). Attempts to synthesize the iron derivatives of *meta*- and *para*-pyridylethynyles under described conditions^62^ were also unsuccessful; the iron acetylide compounds were obtained in very low yields (less than 5%) although no formation of the side binuclear FePd products was observed in these cases.

For that reasons, we became interested in developing a simple and reliable synthetic approach to σ-acetylide iron complexes 1–3 based on the Pd/Cu-catalyzed cross-coupling reactions. We proposed that the inability to obtain the pyridylethynyl iron complexes 1–3 from Cp(CO)_2_FeI and ethynylpyridines under the Oshima conditions was primarily caused by a suppression of the formation and transfer of pyridylethynyl moieties to the palladium atom (Pd/Cu-catalyzed reactions) or of the formation of this moiety at the Fe–Pd center from the π-coordinated alkyne (Cu-free reaction). Here we report on the cross-coupling of terminal ethynylpyridines and (trimetylsilyl)ethynylpyridines with cyclopentadienyliron dicarbonyl iodide under different Pd/Cu-catalyzed conditions to obtain the target iron pyridylethynyl complexes.

## Results and discussion

### Development of Pd/Cu-catalyzed approaches to the synthesis of Cp(CO)_2_Fe–C≡C–(n-C_5_H_4_N) (n = *ortho* (1), *meta* (2), *para* (3)) and Cp(CO)_2_Fe–C≡C–(4-C_6_H_3_N_2_S) (5)

Following on from the hypothesized reasons of the inability to obtain the ethynylpyridyl iron complexes Cp(CO)_2_Fe–C≡C–(n-C_5_H_4_N) (n = *ortho* (1), *meta* (2), *para* (3)), we realized that facilitation of the transmetallation step (the formation and the transfer of acetylide species) in Pd/Cu-catalyzed reaction between Cp(CO)_2_FeI and ethynylpyridines should allow the preparation of the desired products 1–3. Therefore, two approaches were proposed to solve this problem: (i) the use of (trimethylsilyl)ethynylpyridines that in the presence of fluoride ion generate anionic pentacoordinate silicate species [Me_3_Si(F)–C≡C–Pyr]^−^, in which the alkynyl group is more nucleophilic and should be smoothly transferred to a palladium catalyst (Hiyama coupling);^64^ (ii) the application of stronger base than secondary and tertiary amines such as 1,8-diazabicyclo[5.4.0]undec-7-ene (DBU) to facilitate the acetylide species formation in Pd/Cu-catalyzed reactions of Cp(CO)_2_FeI with terminal ethynylpyridines. Our preliminary experiments suggested that both approaches can be used.

The palladium(ii) complexes bis(triphenylphosphine)palladium(ii) dichloride PdCl_2_(PPh_3_)_2_ and bis(acetonitrile)palladium dichloride PdCl_2_(NCMe)_2_, as well as tris(dibenzylideneacetone)dipalladium(0) Pd_2_(dba)_3_ were tested as catalysts in this study. However, in our firsts experiments an application of PdCl_2_(PPh_3_)_2_ as catalyst was found to results in an unexpected substitution of CO ligands by PPh_3_ in [Cp(CO)_2_Fe] fragments to give the side-products Cp(CO)(PPh_3_)FeI and Cp(CO)(PPh_3_)Fe–C≡C–(2-C_5_H_4_N), thereby making the separation of the reaction mixture more difficult. Therefore, in our following experiments we applied only such palladium catalysts that do not contain ligands capable to substitute carbonyl groups in the initial and the target compounds, namely Pd_2_(dba)_3_ and PdCl_2_(NCMe)_2_. The influence of the presence of CuI cocatalyst on the reaction outcome was also examined.

The application of the “(trimethylsilyl)ethynylpyridine” approach resulted in only moderate yields (up to 60%) of the target complexes 1–3; the radical dimerization product [Cp(CO)_2_Fe]_2_ (in 23–37% yield) and the corresponding 1,4-di(*n*-pyridyl)buta-1,3-diynes^65^ (trace amounts) were also obtained. The nature of the palladium catalyst (PdCl_2_(NCMe)_2_ or Pd_2_(dba)_3_) didn't affect the yields of 1–3 here, as well as the presence of CuI as cocatalyst (Table 1). However, a source of tetrabutylammonium fluoride is important; the coupling reactions of Cp(CO)_2_FeI and [(trimethylsilyl)ethynyl]pyridines performed with tetrabutylammonium fluoride trihydrate gave only half of the pyridylethynyl complex's yield obtained in the reactions with 1 M solution of TBAF in THF.

**Table 1:** Coupling reactions of cyclopentadienyliron dicarbonyl iodide and [(trimethylsilyl)ethynyl]pyridines^a^

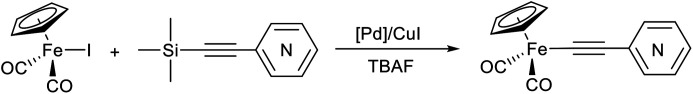							
#	Pyr	[Pd]	CuI, mol%	Conditions	Conversion^b^, %	Yield of products, %	
						Target complex	Byproducts
1	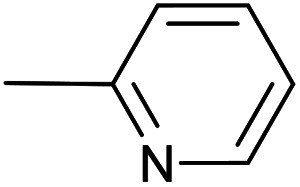	PdCl_2_(PPh_3_)_2_, 10 mol%;	20	40 °C; 120 min	70	1 – 58%	[Cp(CO)_2_Fe]_2_ (3%); Cp(CO)(PPh_3_)FeI (3%); [–C≡C–(2-C_5_H_4_N)]_2_ (5%); Cp(CO)(PPh_3_)Fe–C≡C–(2-C_5_H_4_N) (3%)
2		Pd_2_(dba)_3_, 10 mol%	20	36 °C; 90 min	98	1 – 58%	[Cp(CO)_2_Fe]_2_ (32%); [–C≡C–(2-C_5_H_4_N)]_2_ (2%)
3		Pd_2_(bda)_3_, 10 mol%	—	36 °C; 90 min	98	1 – 60%	[Cp(CO)_2_Fe]_2_ (28%); [-C≡C–(2-C_5_H_4_N)]_2_ (5%)
4		Pd_2_(bda)_3_, 2 mol%	—	60 °C; 90 min	97	1 – 44%	[Cp(CO)_2_Fe]_2_ (37%)
5		PdCl_2_(NCMe)_2_, 10 mol%	20	36 °C; 90 min	95	1 – 58%	[Cp(CO)_2_Fe]_2_ (27%); [–C≡C–(2-C_5_H_4_N)]_2_ (3%)
6		PdCl_2_(NCMe)_2_, 5 mol%	—	50 °C; 90 min	93	1 – 61%	[Cp(CO)_2_Fe]_2_ (23%)
7	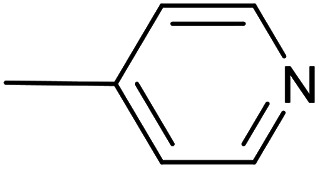	Pd_2_(bda)_3_, 10 mol%	20	36 °C; 90 min	96	3 – 49%	[Cp(CO)_2_Fe]_2_ (32%); [–C≡C–(4-C_5_H_4_N)]_2_ (6%)
8		Pd_2_(bda)_3_, 10 mol%	—	36 °C; 90 min	98	3 – 54%	[Cp(CO)_2_Fe]_2_ (34%); [–C≡C–(4-C_5_H_4_N)]_2_ (5%)
9		PdCl_2_(NCMe)_2_, 10 mol%	20	36 °C; 90 min	97	3 – 44%	[Cp(CO)_2_Fe]_2_ (30%); [–C≡C–(4-C_5_H_4_N)]_2_ (3%)
10		PdCl_2_(NCMe)_2_, 5 mol%	—	50 °C; 90 min	92	3 – 40%	[Cp(CO)_2_Fe]_2_ (37%)
11	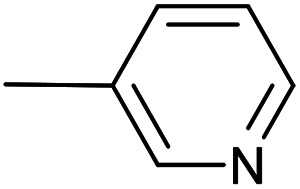	Pd_2_(bda)_3_, 10 mol%	—	36 °C; 90 min	93	2 – 56%	[Cp(CO)_2_Fe]_2_ (27%); [–C≡C–(3-C_5_H_4_N)]_2_ (1%)
12		PdCl_2_(NCMe)_2_, 5 mol%	—	50 °C; 90 min	95	2 – 48%	[Cp(CO)_2_Fe]_2_ (36%)

a All reactions were carried out with use of 1 equiv. Cp(CO)_2_FeI, 1.5 equiv. Me_3_Si–C≡C–(n-C_5_H_4_N), in the presence of 1.5 equiv. TBAF (1 M in THF) in THF.

b Conversion was calculated from the amount of unreacted Cp(CO)_2_FeI recovered by column chromatography.

The “ethynylpyridine” approach was found to be much more effective, the complexes 1–3 were obtained in yields from 67 to 96%. The best catalytic system in this case was PdCl_2_(NCMe)_2_/CuI, with which iron-ethynylpyridine coupling proceeds efficiently at room temperature in 20 minutes (2 mol% of Pd catalysts and 20 mol% of CuI) or at 60 °C in 30 minutes with reduced catalysts loadings (1 and 5 mol%, respectively) (Table 2).

**Table 2:** Pd(ii)/CuI- and Pd(ii)-catalyzed coupling reactions of cyclopentadienyliron dicarbonyl iodide and ethynylpyridines^a^

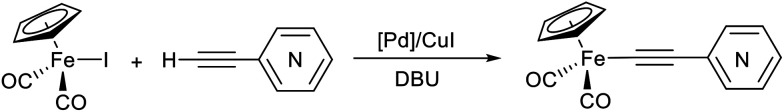							
#	Pyr	[Pd]	CuI, mol%	Conditions	Conversion, %	Yield of products, %	
						Target complex	Byproducts
1	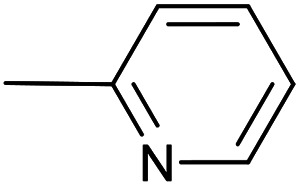	PdCl_2_(PPh_3_)_2_, 10 mol%;	20	40 °C; 30 min	86	1 – 66%	Cp(CO)(PPh_3_)FeI (3%); Cp(CO)(PPh_3_)Fe–C≡C–(2-C_5_H_4_N) (8%)
2		PdCl_2_(NCMe)_2_, 10 mol%	20	24 °C; 20 min	98	1 – 91%	[Cp(CO)_2_Fe]_2_ (5%);
3		PdCl_2_(NCMe)_2_, 2 mol%	20	24 °C; 20 min	97	1 – 94%	[Cp(CO)_2_Fe]_2_ (2%);
4		PdCl_2_(NCMe)_2_, 1 mol%	5	60 °C; 30 min	98	1 – 92%	[Cp(CO)_2_Fe]_2_ (4%);
5		PdCl_2_(NCMe)_2_, 5 mol%	—	60 °C; 90 min	29	1 – 15%	[Cp(CO)_2_Fe]_2_ (9%); 4 – 3% [Fe], 55% [Pd]^b^
6	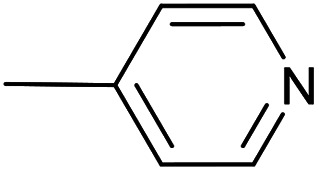	PdCl_2_(NCMe)_2_, 10 mol%	20	24 °C; 20 min	97	3 – 91%	—
7		PdCl_2_(NCMe)_2_, 2 mol%	20	24 °C; 20 min	96	3 – 92%	—
8		PdCl_2_(NCMe)_2_, 1 mol%	5	60 °C; 30 min	99	3 – 96%	—
9		PdCl_2_(NCMe)_2_, 5 mol%	—	60 °C; 90 min	75	3 – 67%	[Cp(CO)_2_Fe]_2_ (7%)
10	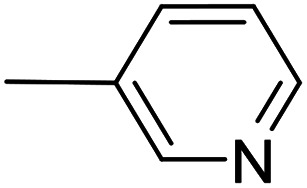	PdCl_2_(NCMe)_2_, 10 mol%	20	24 °C; 20 min	99	2 – 83%	[Cp(CO)_2_Fe]_2_ (13%)
11		PdCl_2_(NCMe)_2_, 2 mol%	20	24 °C; 20 min	98	2 – 93%	—
12		PdCl_2_(NCMe)_2_, 1 mol%	5	60 °C; 30 min	99	2 – 95%	—
13		PdCl_2_(NCMe)_2_, 5 mol%	—	60 °C; 90 min	74	2 – 66%	[Cp(CO)_2_Fe]_2_ (6%)

a All reactions were carried out with use of 1 equiv. Cp(CO)_2_FeI, 1.5 equiv. H–C≡C–(n-C_5_H_4_N), in the presence of 1.5 equiv. DBU in THF.

b Isolated yield of 4 based on Cp(CO)_2_FeI and PdCl_2_(NCMe)_2_ consumption.

The presence of CuI in these conditions is important for high-yield synthesis of 1–3. The pyridylethynylation smoothly proceeds only for the Pd(ii)/CuI-catalyzed reactions, whereas in the absence of CuI cocatalyst (Table 2, entries 5, 9 and 13) the conversion and the yield of corresponding σ-pyridylethynyl complex decreased, but the yield of the dimerization product [Cp(CO)_2_Fe]_2_ increased. Moreover, in these conditions the reaction with 2-ethynylpyridine gave μ-pyridylvinylidene complex 4 as a side product in 4% yield.

Similar results were obtained for the reactions between Cp(CO)_2_FeI and ethynylpyridines catalyzed by the zero-valent palladium complex Pd_2_(dba)_3_ (Table 3). When the Pd_2_(dba)_3_/CuI pair was applied, the target pyridylethynyl complexes 1–3 were produced in high yields (84, 86 and 87% for 1, 2 and 3, respectively) and the yields of the dimerization product [Cp(CO)_2_Fe]_2_ were in a range of 6–8%. However, under copper-free conditions here, the yield of the target complexes decreased by about 20%, while the yield of [Cp(CO)_2_Fe]_2_ increased to 20% and, also, μ-pyridylvinylidene complex 4 was obtained in the case of the reaction of Cp(CO)_2_FeI and H–C≡C–(2-C_5_H_4_N) (Table 3, entries 2 and 4). Thus, the binuclear complex 4 is also produced under the conditions of catalytic formation of complex 1. It should be noted that for the reactions between Cp(CO)_2_FeI and ethynylpyridines we decided to apply the same temperature and Pd_2_(dba)_3_ loadings that gave the highest yields of 1–3 in the ethynylation of Cp(CO)_2_FeI with [(trimethylsilyl)ethynyl]pyridines (entries 3, 8, 11 in Table 1).

**Table 3:** Pd(0)/CuI- and Pd(0)-catalyzed coupling reactions of cyclopentadienyliron dicarbonyl iodide and ethynylpyridines^a^

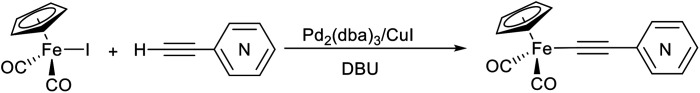							
#	Pyr	[Pd], mol%	CuI, mol%	Conditions	Conversion, %	Yield of products, %	
						Target complex	Byproducts
1	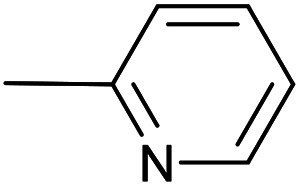	10	20	36 °C; 90 min	96	1 – 84%	[Cp(CO)_2_Fe]_2_ (8%); [–C≡C–(2-C_5_H_4_N)]_2_ (3%)
2		10	—	36 °C; 90 min	95	1 – 64%	[Cp(CO)_2_Fe]_2_ (17%); 4 – 7% [Fe], 72% [Pd]^b^
3	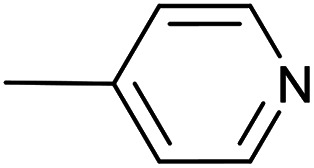	10	20	36 °C; 90 min	95	3 – 86%	[Cp(CO)_2_Fe]_2_ (6%); [–C≡C–(4-C_5_H_4_N)]_2_ (2%)
4		10	—	36 °C; 90 min	96	3 – 67%	[Cp(CO)_2_Fe]_2_ (20%); [–C≡C–(4-C_5_H_4_N)]_2_ (4%)
5	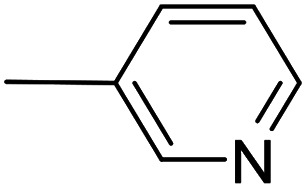	10	20	36 °C; 90 min	96	2 – 87%	[Cp(CO)_2_Fe]_2_ (7%); [–C≡C–(3-C_5_H_4_N)]_2_ (3%)

a All reactions were carried out with use of 1 equiv. Cp(CO)_2_FeI, 1.5 equiv. H–C≡C–(n-C_5_H_4_N), in the presence of 1.5 equiv. DBU in THF.

b Isolated yield of 4 based on Cp(CO)_2_FeI and Pd_2_(dba)_3_ consumption.

Therefore, all reactions given in Table 3 were conducted at 36 °C with 10 mol% of Pd_2_(dba)_3_.

**Scheme 2:**
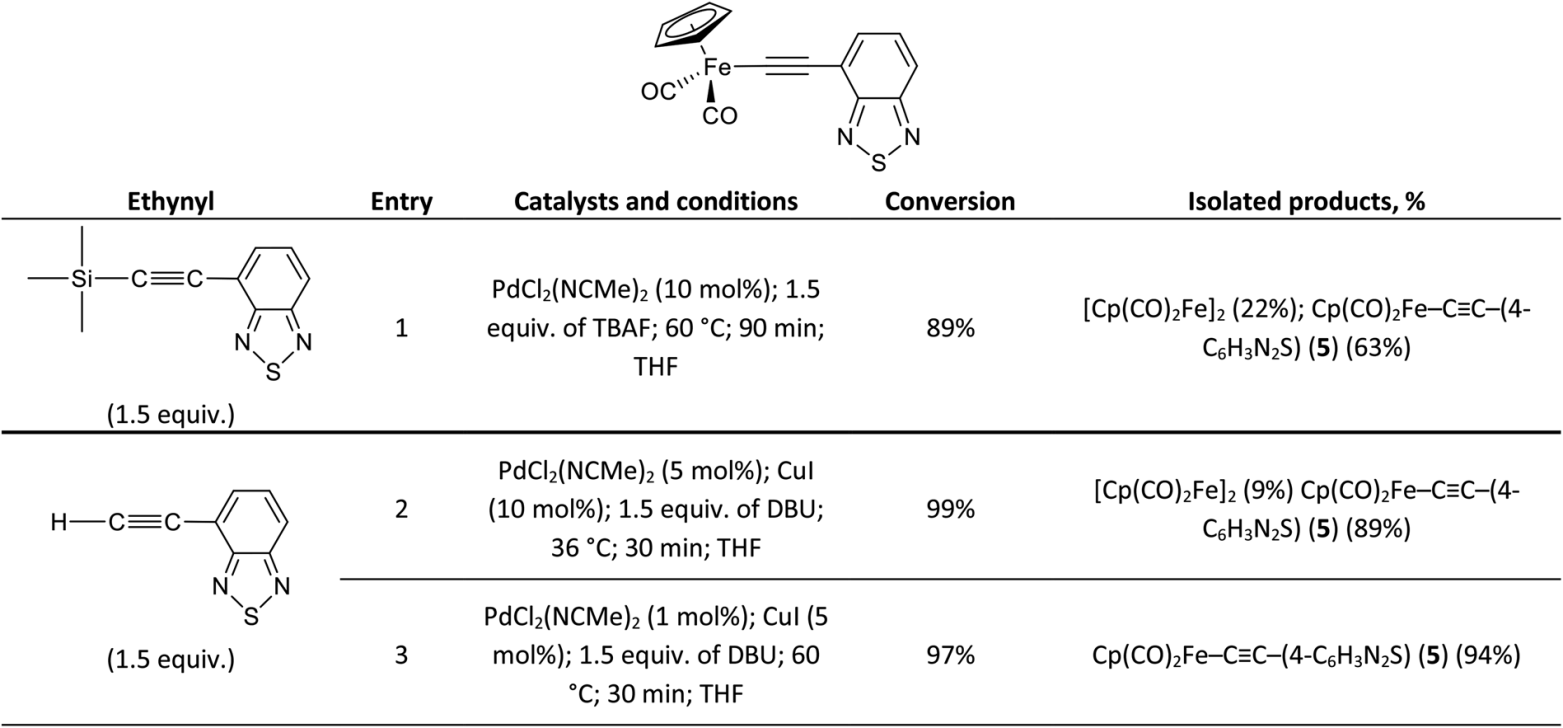
The results on the Pd/Cu-catalyzed synthesis of Cp(CO)_2_Fe–C≡C–(4-C_6_H_3_N_2_S) (5).

To emphasize the applicability of the Pd/Cu-catalyzed pyridylethynylation of Cp(CO)_2_FeI to the synthesis of similar compounds, we tried to prepare the corresponding σ-4-benzothiadiazolylethynyl complex Cp(CO)_2_Fe–C≡C–(4-C_6_H_3_N_2_S) (5). Following the developed strategies, we investigated the coupling reactions of Cp(CO)_2_FeI with 4-(trimethylsilyl)ethynyl-2,1,3-benzothiadiazole and 4-ethynyl-2,1,3-benzothiadiazole. In all experiments performed using both approaches complex 5 was obtained. However, its highest yield 94% was achieved only in the coupling of Cp(CO)_2_FeI and H–C≡C–(4-C_6_H_3_N_2_S) at 60 °C using 1 mol% of PdCl_2_(NCMe)_2_ and 5 mol% of CuI (Scheme 2, entry 3).

### The proposed catalytic cycles

**Scheme 3:**
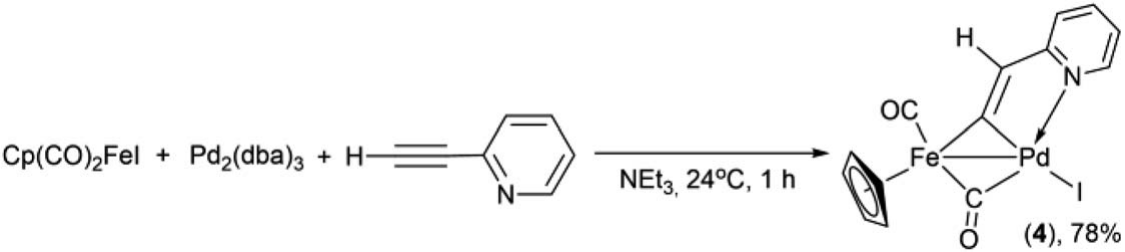
Reaction of high yield synthesis of the binuclear FePd μ-pyridylvinylidene complex 4.

Previously, the Lo Sterzo group clearly demonstrated a close analogy between the Pd-catalyzed carbon–carbon and metal–carbon bond formation mechanisms through reactions between [η^5^-(1-diphenylphosphino-2,4-diphenyl)cyclopentadienyl](CO)_3_MI (M = Mo, W) and PdCl_2_(NCMe)_2_ with a following treatment of the resulting binuclear MPd oxidation addition complexes with 4-nitro-1-[2-(tributhylstannyl)ethynyl]benzene.^57,59^ The mechanism of the Pd/Cu- and Pd-catalyzed pyridylethynylation of cyclopentadienyliron dicarbonyl iodide should be apparently analogous to that of the carbon–carbon coupling reactions. Some additional clues to the mechanisms of the formation of the complexes 1–3, 5 may be provided by a consideration of the reactions between Cp(CO)_2_FeI and 2-ethynylpyridine. The formation of the binuclear FePd μ-pyridylvinylidene complex 4 in the reactions under Cu-free conditions (Table 2 entries 5, 9, 13 and Table 3 entries 2, 4) clearly shows that the reaction pathways leading to 1 and 4 may have common intermediates. Moreover, one can assume that in the reaction performed with the stoichiometric ratios of Pd_2_(dba)_3_, Cp(CO)_2_FeI and H–C≡C–(2-C_5_H_4_N), the ratio of 1 to 4 should change in favor of the latter. Indeed, the reaction of Cp(CO)_2_FeI with 2-ethynylpyridine in the presence of 1 equiv. of Pd_2_(dba)_3_ in triethylamine as a solvent gave complex 4 in yield of 78% (Scheme 3). However, the trace amount (3% yield) of the 2-pyridylethynyl complex 1 was obtained, thereby confirming that the catalytic 2-pyridylethynylation of Cp(CO)_2_FeI occurs even under these conditions, although in a much lesser degree.

**Scheme 4:**
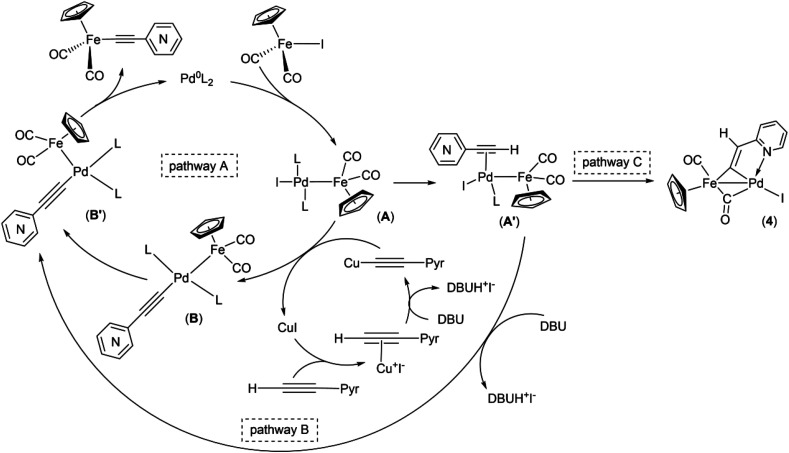
Proposed mechanism of Pd/Cu- and Pd-catalyzed coupling reactions of cyclopentadienyliron dicarbonyl iodide and ethynylpyridines.

Obviously, one of these common intermediates in the reaction pathways to 1 and 4 is binuclear FePd species (Scheme 4, A) formed by an oxidative addition of Cp(CO)_2_FeI to catalytically active Pd^0^ species. In the absence of an efficient transmetallating agent, such as the copper acetylide or anionic pentacoordinate silicate adduct [Me_3_Si(F)–C≡C–(2-C_5_H_4_N)]NBu_4_, a π-coordination of the alkyne to A can occur resulting in complex A′. Without a strong base A′ rearrange into the μ-pyridylvinylidene complex 4. To reveal the mechanism of this process, an additional study is needed, since there are several possible reaction pathways for its proceeding. It is worth noting that the examples of the acetylene-to-vinylidene tautomerism involving two adjacent metal centers are known and described in several works,^66–70^ but they are much less studied in contrast to the well-understood acetylene-to-vinylidene rearrangement mediated by a single metal center.^71,72^ It is also worth to note that the *ortho*-position of the nitrogen atom in 2-ethynylpyridine plays an important role in the process of formation of the binuclear FePd μ-pyridylvinylidene complex 4, as the Pd-catalyzed reactions of Cp(CO)_2_FeI with 3- and 4-ethynylpyridines simply resulted in a decrease of yields of 2 and 3, and didn't give any binuclear products (Table 2 entries 9 and 13).

Taking into account the data obtained and the known mechanistic aspects of the cross-coupling reactions, a noncontradictory mechanistic scheme of the pyridylethynylation of cyclopentadienyliron dicarbonyl iodide was proposed (Scheme 4), which explains the reaction pattern and the possibility of alternative reaction pathway. For the Pd/Cu-catalyzed reactions (pathway A), the first step of the catalytic cycle would be an oxidative addition of the Cp(CO)_2_FeI to the catalytically active Pd^0^L_2_ species to give a binuclear FePd intermediate A. A subsequent transmetallation, where transfer of the pyridylacetylide moiety from the Cu-acetylide formed in the copper-cycle to the FePd complex A led to a complex B, which undergoes *trans* to *cis* isomerization to give a complex B′, a following reductive elimination of the latter results in the Fe–C cross-coupling products Cp(CO)_2_Fe–C≡C–(n-C_5_H_4_N) (n = *ortho* (1), *meta* (2), *para* (3)). The initial oxidative addition and the final reductive elimination steps of a mechanism for the Cu-free reaction (pathway B) are the same as for the Pd/Cu-catalyzed one. However, an alternative ligand L substitution in the intermediate A by the alkyne can take place resulting in π-alkyne complex A′. Its subsequent deprotonation then occurs to give the complex B′. The generation of the complex 4 in this context can be considered as a side pathway C of the Pd-catalyzed reaction between Cp(CO)_2_FeI and 2-ethynylpyridine that is facilitated by the absence of transmetallating agents or appropriate base, and *ortho*-position of the nitrogen atom in the pyridine ring of the alkyne. The catalytic cycle for the complex 5 formation should be the same as for 1–3.

**Scheme 5:**
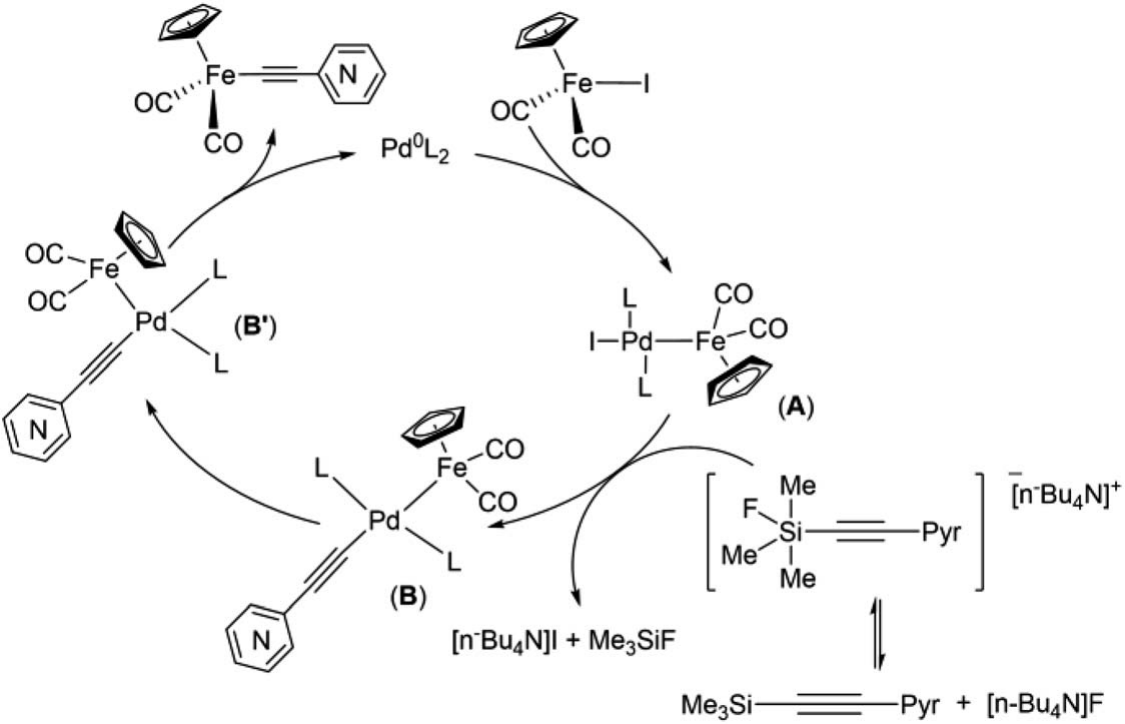
Proposed mechanism of Pd-catalyzed coupling reactions of cyclopentadienyliron dicarbonyl iodide and [(trimethylsilyl)ethynyl]pyridines.

The results obtained in the case of the “(trimethylsilyl)ethynylpyridine” demonstrated that pyridylethynylation of Cp(CO)_2_FeI should proceed according to the reaction pathway commonly accepted for the Hiyama coupling, *i.e.* transfer of the pyridylacetylide moiety to the FePd complex A should goes through anionic pentacoordinate silicate species^64^ (Scheme 5).

### Characterization of the complexes

The IR and the ^1^H and ^13^C{^1^H} NMR data for the complexes 1–3 and 5 were obtained. The NMR signals were assigned on the basis of ^1^H–^13^C correlations measured through HSQC and HMBC experiments, respectively (Table 4). The structure of the complexes can be deduced from the combined NMR and IR data.

**Table 4:** IR and NMR (*δ*, ppm [*J*, Hz]) spectroscopic data for the Cp(CO)_2_Fe–C≡C–(n-C_5_H_4_N) [n = *ortho* (1), *meta* (2), *para* (3)] and Cp(CO)_2_Fe–C≡C–(4-C_6_H_3_N_2_S) (5)

	NMR (*δ*, ppm [*J*, Hz], CD_2_Cl_2_)					IR, cm^−1^ (CH_2_Cl_2_)
	^1^H	^13^C				
	C_5_H_5_	–C_α_≡C_β_–R		C_5_H_5_	CO	
		C_α_	C_β_			
1	5.14	116.7	95.4	85.4	212.2	2112 (*ν*_C≡C_), 2043, 1997 (*ν*_CO_)
2	5.13	112.3	95.7	85.3	212.4	2111 (*ν*_C≡C_), 2044, 1996 (*ν*_CO_)
3	5.13	114.3	102.0	85.6	212.3	2110 (*ν*_C≡C_), 2044, 1998 (*ν*_CO_)
5	5.20	112.3	102.9	85.6	212.3	2100 (*ν*_C≡C_), 2042, 1997 (*ν*_CO_)

The IR spectra of 1–3 in CH_2_Cl_2_ solution show two very strong absorptions at about 2043 and 1996 cm^−1^ that correspond to the *ν*(C≡O) stretching modes of carbonyl groups at the iron atom. An additional absorption with strong intensity at about 2111 cm^−1^ is attributed to the *ν*(C≡C) stretching frequencies of the alkynyl ligand. The *ν*(CO) and the *ν*(C≡C) frequencies of the complex 5 with σ-4-ethynyl-2,1,3-benzothiadiazole ligand are insignificantly shifted to low-frequency in comparison with 1–3.

The ^13^C nuclei of α- and β-alkynyl carbons of 1–3 and 5 resonate in the regions *δ* 112–117 and 95–103 ppm, respectively. It is noteworthy that these signals slightly vary with the nature of the substituents in σ-alkynyl ligands. The downfield shift of C_α_ nuclei by approximately 2 ppm is observed on moving from the *meta*- to *para*- and to *ortho*-pyridylethynyl iron complexes. However, this trend doesn't hold for the C_β_ nuclei, here only the signal of 3 is downfield shifted by 7 ppm compared with those of the 1 and 2. These correlations are apparently due to changes in π-electron distribution induced by the electronic effect of the pyridyl fragments. However, these effects can't be reduced to a single parameter (like only inductive or mesomeric effects), as was showed by Claude Lapinte team for cationic complexes of the type [Cp*(dppe)Fe–C≡C–(*n*-C_5_H_4_N)][PF_6_] (*n* = 2, 3, 4).^73^ At the same time, the ^13^C chemical shifts of the cyclopentadienyl and carbonyl groups coordinated to the iron atom are almost independent of their nature (Table 4). The presence of the pyridyl substituents in 1–3 are indicated by signals between 7.02 and 8.50 ppm in ^1^H NMR spectra, and between 119 and 152 ppm in ^13^C NMR spectra of the complexes. The carbon and hydrogen atoms of the 2,1,3-benzothiadiazole group in 5 resonate in the regions *δ* 118–156 and *δ* 7.48–7.76 ppm in ^13^C and ^1^H NMR spectra, respectively. So, overall the IR and NMR spectra parameters of 1–3 and 5 are similar to those found for analogous cyclopentadienyliron dicarbonyl complexes with different σ-alkynyl ligands.^34,54,62^

**Fig. 1:**
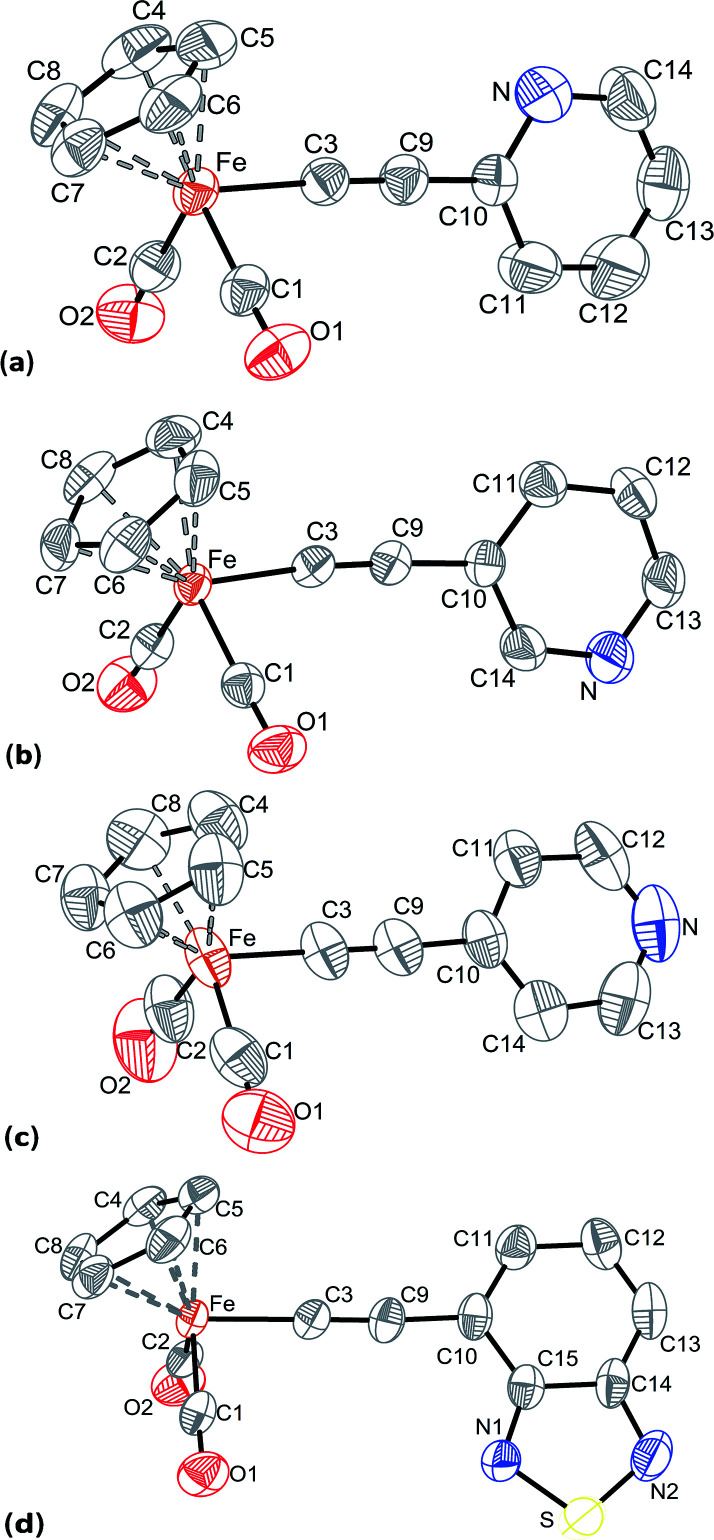
Molecular structures of 1 (a), 2 (b), 3 (c), and 5 (d) with the thermal ellipsoids set at the 50% probability level.

The molecular structures of the complexes Cp(CO)_2_Fe–C≡C–(2-C_5_H_4_N) (1), Cp(CO)_2_Fe–C≡C–(3-C_5_H_4_N) (2), Cp(CO)_2_Fe–C≡C–(4-C_5_H_4_N) (3), and Cp(CO)_2_Fe–C≡C–(4-C_6_H_3_N_2_S) (5) were solved on the basis of X-ray diffractometry data. Suitable crystals of 1–3 and 5 were grown from dichloromethane/hexane mixtures. The views of the structures are shown in Fig. 1, selected bond lengths and angles are given in Table 5. The crystal data and refinement parameters are included in the ESI.†

**Table 5:** The selected bond distances (Å) and angles (°) for the complexes 1–3 and 5

	1	2	3	5
**Distances, Å**				
Fe–C3	1.9159(17)	1.919(2)	1.906(3)	1.917(3)
C3–C9	1.204(2)	1.200(3)	1.203(5)	1.208(4)
C9–C10	1.436(2)	1.437(3)	1.423(4)	1.435(4)
Fe–Cp	1.7223(8)	1.7200(11)	1.7186(19)	1.7222(16)

**Bond angles, °**				
Fe–C3–C9	175.94(16)	174.3(2)	179.4(4)	178.6(3)
C3–C9–C10	176.54(19)	176.8(3)	177.7(5)	174.6(4)
C3–Fe–C1	89.26(8)	86.8(1)	89.68(17)	88.80(15)
C3–Fe–C2	87.11(8)	89.4(1)	89.07(16)	89.56(14)

**Angles between planes, °**				
η^5^-Cp:ring^a^	84.51(7)	84.58(8)	68.64(14)	71.22(12)
(O1–C1–Fe–C2–O2):ring^a^	81.38(6)	80.97(7)	88.27(8)	81.40(8)

a The angles between appropriate plane and plane of the substituent's ring (pyridine in 1–3 and benzothiadiazole in 5) of the σ-alkynyl ligands are given.

The proximity of crystals 1 and 2 cell parameters (Table S3†) and small differences in the sequence of atoms in their molecules suggest that there are small differences in a packing of molecules. Indeed, the structures 1 and 2 are mirror-like each other, and the mirror plane is perpendicular to the cell axis *b*. The iron atom in 1–3 and 5 is coordinated by a cyclopentadienyl ring in η^5^-fashion, two terminal CO ligands, and by σ-alkynyl ligand to adopt a typical pseudo-octahedral geometry. The bond lengths and angles in 1–3 and 5 are close to each other and to those found in the known complexes of the type Cp(CO)_2_Fe–C≡C–R.^74–77^ The differences in geometry of the complexes' molecules are associated with the position of C≡C–R moiety relative to the iron fragment. The pyridylethynyl ligands in 1 and 2, and the 4-ethynyl-2,1,3-benzothiadiazolyl ligand in 5 lean slightly toward one of the carbonyl group of the iron atom in such a way that the planes between ring of the substituent and [Fe(CO)_2_] fragment exhibit angles about 81°. At the same time, in the complex 3 the *para*-pyridylethynyl ligand is almost perpendicular to the plane formed by two carbonyl ligands and the iron atom. Moreover, the angles between the planes of the substituent's ring in the σ-alkynyl and the cyclopentadienyl ligands are about 70° in 3 and 5, whereas the analogous angles in 1 and 2 are *ca.* 85°. It is also of interest that the pyridyl substituents in 1 and 2 differ in their orientations: when the nitrogen atom in 1 is oriented to Cp ring, that in the complex 2 the nitrogen is placed opposite, *i.e.* oriented to carbonyl groups.

## Conclusions

In this paper, two approaches were developed for the pyridylethynylation of cyclopentadienyliron dicarbonyl iodide based on Pd/Cu- and Pd-catalyzed Fe–C coupling of (trimethylsilyl)ethynylpyridines and ethynylpyridines with Cp(CO)_2_FeI. Although these two approaches differ only in the nature of species participating in the transmetallation step (transfer of the pyridylacetylide moiety to catalytically active species), the second “ethynylpyridine” approach was found to be much more effective compared with the first “(trimethylsilyl)ethynylpyridine” one. For example, the σ-pyridylethynyl iron complexes Cp(CO)_2_Fe–C≡C–(n-C_5_H_4_N) (n = *ortho* (1), *meta* (2), *para* (3)) were obtained in highest yields (about 95%) in the reaction of ethynylpyridines with Cp(CO)_2_FeI in THF in the presence of 1,8-diazabicyclo[5.4.0]undec-7-ene as a base upon the catalysis with 1 mol% of PdCl_2_(NCMe)_2_ and 5 mol% of CuI and heating at 60 °C for 30 minutes. In case of the Cu-free coupling between Cp(CO)_2_FeI and 2-ethynylpyridine, there was an additional side-reaction, which resulted in traces of the binuclear complex [Cp(CO)Fe{μ_2_-η^1^(C_α_):η^1^(C_α_)-κ^1^(N)-C_α_=C_β_(H)(2-C_5_H_4_N)}(μ-CO)PdI] (4). The oxidative addition species Cp(CO)_2_Fe-[PdL_2_]-I (A in Scheme 4) are supposed to be a key intermediate in the catalytic cycle of the σ-alkynyl complexes formation, however in the absence of effective transmetallating agents or appropriate base A is assumed to become a precursor to 4.

The developed methodology can be extended to the synthesis of other σ-alkynyl complexes of iron. So, the coupling of cyclopentadienyliron dicarbonyl iodide and 4-ethynyl-2,1,3-benzothiadiazole under the same condition as above was found to proceed efficiently resulting in the complex Cp(CO)_2_Fe–C≡C–(4-C_6_H_3_N_2_S) (5). The spectroscopic and structural features of 1–3, 5 were described.

## Experimental

### General considerations

All operations and manipulations were carried out under an argon atmosphere. Solvents (dichloromethane, petroleum ether, ethyl acetate, hexane, triethylamine) were purified by distillation from the appropriate drying agents and stored under argon. THF was dried by refluxing over sodium/benzophenone ketyl and freshly distilled prior to use. The course of reactions was monitored by TLC on Silica gel (Alu foils, Sigma-Aldrich) and IR spectroscopy. Neutral silica gel (silica 60, 0.2–0.5 mm, Macherey-Nagel) was used for column chromatography. Pd(Cl)_2_(PPh_3_)_2_,^78^ Pd(Cl)_2_(NCMe)_2_,^79^ Pd_2_(dba)_3_·CHCl_3_,^80^ and Cp(CO)_2_FeI^81^ were prepared according to literature procedures. *Ortho*-, *meta*-, *para*-[(trimethylsilyl)ethynyl]pyridines^82^ and 4-(trimethylsilyl)ethynyl-2,1,3-benzothiadiazole^83^ were prepared from ethynyltrimethylsilane. *Ortho*- and *para*-ethynylpyridines were synthesised from 2-methyl-4-(*n*-pyridyl)but-3-yn-2-ols by elimination of acetone, according to published procedures.^65^*Meta*-ethynylpyridine and 4-ethynyl-2,1,3-benzothiadiazole were obtained by desilylation of *meta*-[(trimethylsilyl)ethynyl]pyridine^84^ and 4-(trimethylsilyl)ethynyl-2,1,3-benzothiadiazole.^83^ Tetrabutylammonium fluoride solution (1 M solution in THF, Aldrich), 1,8-diazabicyclo[5.4.0]undec-7-ene (Aldrich), and catalyst CuI (“Vekton-M” Ltd.) were purchased and used directly. The synthetic procedures for the preparation of 1–3 and 5 providing the highest yields are described in the experimental part (Table 2, entries 4, 8, and 12; Scheme 2, entry 3), the detailed isolation procedures and characterization of the products can be found in the ESI.†

Physical-chemical characteristics were obtained in the Krasnoyarsk Regional Centre of Research Equipment, Siberian Branch of the Russian Academy of Sciences. The IR spectra were recorded on the Shimadzu IR Tracer-100 spectrometer (Japan). ^1^H, ^13^C{^1^H}, ^31^P{^1^H}, HSQC, and HMBC NMR spectra were obtained using NMR spectrometer AVANCE III 600 (Bruker, Germany). Chemical shifts are reported in ppm units referenced to residual solvent resonances for ^1^H, ^13^C{^1^H}, HSQC, and HMBC spectra or to an external 85% H_3_PO_4_(aq) standard for ^31^P{^1^H} spectra. The X-ray data for 1–3 and 5 were obtained with the Smart Photon II diffractometer (Bruker AXS, Germany).

### Synthesis of Cp(CO)_2_Fe–C≡C–(2-C_5_H_4_N) (1)

Cyclopentadienyliron dicarbonyl iodide (315 mg, 1.036 mmol) and 2-ethynylpyridine (0.155 mL, 158 mg, 1.534 mmol) were dissolved in freshly distilled THF (10 mL), then DBU (0.23 mL, 234 mg, 1.539 mmol), PdCl_2_(NCMe)_2_ (3 mg, 0.012 mmol), and CuI (10 mg, 0.052 mmol) were added. The reaction mixture was stirred at 60 °C for 30 minutes, and then was evaporated to dryness; the residue was dissolved in dichloromethane and passed through a pad (0.5 cm) of silica gel by using ethyl acetate as an eluent. The filtrate was concentrated *in vacuo* and chromatographed on silica gel (9 × 2 cm). A dark-yellow fraction containing the σ-alkynylpyridine iron complex was eluted with petroleum ether-ethyl acetate (3 : 7) mixture and subsequently with ethyl acetate. The complex Cp(CO)_2_Fe–C≡C–(2-C_5_H_4_N) (1) was isolated as brown-yellow solid after evaporation of the solvent (266 mg, 0.953 mmol, 92%). ^1^H NMR (CD_2_Cl_2_, 25 °C) *δ*, ppm [*J*, Hz]: 5.14 (s, 5H, C_5_***H***_5_); 7.02 (ddd, 1H, ^3^*J*_HH_ = 7.6 Hz, ^3^*J*_HH_ = 5.3 Hz, ^4^*J*_HH_ = 0.8 Hz, ***H***_*para*_ of (2-Pyr) (H^4^)); 7.22 (d, 1H, ^3^*J*_HH_ = 8.0 Hz, ***H***_*ortho*_ of (2-Pyr) (H^6^)); 7.53 (td, 1H, ^3^*J*_HH_ = 7.7 Hz, ^4^*J*_HH_ = 1.6 Hz, ***H***_*meta*_ of (2-Pyr) (H^5^)); 8.40 (d, 1H, ^3^*J*_HH_ = 5.3 Hz, N–C–***H***_*meta*_ of ≡C^2^(2-Pyr) (H^3^)). ^13^C{^1^H} NMR (CD_2_Cl_2_, 25 °C) *δ*, ppm [*J*, Hz]: 85.4 (s, ***C***_5_H_5_); 95.4 (s, ≡***C***^2^–); 116.7 (s, –***C***^1^≡); 119.9 (s, ***C***_*meta*_ of (2-Pyr (C^5^))); 125.9 (s, N–***C***_*meta*_ of (2-Pyr) (C^6^)); 135.3 (s, ***C***_*para*_ of (2-Pyr) (C^4^)); 145.8 (s, ***C***_*ipso*_ of (2-Pyr));149.0 (s, ***C***_*ortho*_ of (2-Pyr) (C^3^)); 212.2 (s, 2Fe–***C***O). IR (CH_2_Cl_2_*ν*/cm^−1^): 2112s (*ν*_C≡C_), 2043vs, 1997vs (*ν*_CO_), 1582m, 1556w, 1460m (*ν*_C=C_ and *ν*_C=N_). IR (KBr *ν*/cm^−1^): 2108s (*ν*_C≡C_), 2034vs, 1982vs (*ν*_CO_), 1582m, 1556w, 1457m (*ν*_C=C_ and *ν*_C=N_). Anal. found: C, 60.41%; H, 3.26%; N, 5.01%. Calc. for C_14_H_9_FeNO_2_ (279): C, 60.25%; H, 3.25%; N, 5.02%.

### Synthesis of Cp(CO)_2_Fe–C≡C–(3-C_5_H_4_N) (2)

Following the procedure described for preparation of 1, complex Cp(CO)_2_Fe–C≡C–(3-C_5_H_4_N) (2) was obtained with 95% yield (218 mg, 0.781 mmol) from cyclopentadienyliron dicarbonyl iodide (250 mg, 0.822 mmol) and 3-ethynylpyridine (127 mg, 1.223 mmol) with the use of THF (8 mL), DBU (0.18 mL, 183 mg, 1.204 mm), PdCl_2_(NCMe)_2_ (2 mg, 0.008 mmol), and CuI (8 mg, 0.042 mmol). ^1^H NMR (CD_2_Cl_2_, 25 °C) *δ*, ppm [*J*, Hz]: 5.13 (s, 5H, C_5_***H***_5_); 7.14 (dd, 1H, ^3^*J*_HH_ = 7.3 Hz, ^3^*J*_HH_ = 5.0 Hz, ***H***_*meta*_ of (3-Pyr) (H^5^)); 7.54 (d, 1H, ^3^*J*_HH_ = 8.0 Hz, ***H***_*para*_ of (3-Pyr) (H^6^)); 8.30 (d, 1H, ^3^*J*_HH_ = 3.2 Hz, ***H***_*ortho*_ of (3-Pyr) (H^4^)); 8.49 (s, 1H, N–C–***H***_*ortho*_ of (3-Pyr) (H^2^)). ^13^C{^1^H} NMR (CD_2_Cl_2_, 25 °C) *δ*, ppm [*J*, Hz]: 85.3 (s, ***C***_5_H_5_); 95.7 (s, ≡***C***^2^–); 112.3 (s, –***C***^1^≡); 122.4 (s, ***C***_*meta*_ of (3-Pyr) (C^5^)); 124.6 (s, ***C***_*ipso*_ of (3-Pyr)) 137.3 (s, ***C***_*para*_ of (3-Pyr) (C^6^)); 145.3 (s, ***C***_*ortho*_ of (3-Pyr) (C^4^)); 152.1 (s, N–***C***_*ortho*_ of (2-Pyr) (C^2^)) 212.4 (s, 2Fe–***C***O). IR (CH_2_Cl_2_*ν*/cm^−1^): 2111s (*ν*_C≡C_), 2044vs, 1996vs (*ν*_CO_), 1580w, 1561w, 1474w (*ν*_C=C_ and *ν*_C=N_). IR (KBr *ν*/cm^−1^): 2103s (*ν*_C≡C_), 2038vs, 1994vs (*ν*_CO_), 1572w, 1555w, 1473w (*ν*_C=C_ and *ν*_C=N_). Anal. found: C, 60.21%; H, 3.26%; N, 5.00%. Calc. for C_14_H_9_FeNO_2_ (279): C, 60.25%; H, 3.25%; N, 5.02%.

### Synthesis of Cp(CO)_2_Fe–C≡C–(4-C_5_H_4_N) (3)

Similarly, Cp(CO)_2_Fe–C≡C–(4-C_5_H_4_N) (3) was obtained from cyclopentadienyliron dicarbonyl iodide (339 mg, 1.115 mmol) and 4-ethynylpyridine (173 mg, 1.680 mmol) with the use of THF (10 mL), DBU (0.25 mL, 255 mg, 1.678 mmol), PdCl_2_(NCMe)_2_ (3 mg, 0.012 mmol), and CuI (11 mg, 0.052 mmol) was obtained Cp(CO)_2_Fe–C≡C–(4-C_5_H_4_N) (3) (299 mg, 1.072 mmol, 96%). ^1^H NMR (CD_2_Cl_2_, 25 °C) *δ*, ppm [*J*, Hz]: 5.13 (s, 5H, C_5_***H***_5_); 7.11 (dd, 2H, ^3^*J*_HH_ = 5.4 Hz, ***H***_*meta*_ of (4-Pyr)); 8.39 (d, 2H, ^3^*J*_HH_ = 4.7 Hz, ***H***_*ortho*_ of (4-Pyr)).^13^C{^1^H} NMR (CD_2_Cl_2_, 25 °C) *δ*, ppm [*J*, Hz]: 85.6 (s, ***C***_5_H_5_); 102.1 (s, ≡***C***^2^–); 114.2 (s, –***C***^1^≡); 125.5 (s, ***C***_*ortho*_ of (4-Pyr)); 135.3 (s, ***C***_*ipso*_ of (4-Pyr)); 149.3 (s, ***C***_*meta*_ of (3-Pyr)); 212.3 (s, 2Fe–***C***O). IR (CH_2_Cl_2_*ν*/cm^−1^): 2110s (*ν*_C≡C_), 2044vs, 1998vs (*ν*_CO_), 1591s (*ν*_C=C_ and *ν*_C=N_). IR (KBr *ν*/cm^−1^): 2109s (*ν*_C≡C_), 2037vs, 1992vs (*ν*_CO_), 1589s, 1526w, 1487w (*ν*_C=C_ and *ν*_C=N_). Anal. found: C, 60.39%; H, 3.24%; N, 5.04%. Calc. for C_14_H_9_FeNO_2_ (279): C, 60.25%; H, 3.25%; N, 5.02%.

### Synthesis of [Cp(CO)Fe{μ_2_-η^1^(C_α_):η^1^(C_α_)-κ^1^(N)-C_α_=C_β_(H)(2-C_5_H_4_N)}(μ-CO)PdI] (4)

Cyclopentadienyliron dicarbonyl iodide (162 mg, 0.533 mmol) and Pd_2_(dba)_3_·CHCl_3_ (277 mg, 0.268 mmol) were dissolved in triethylamine (8 mL). 2-Ethynylpyridine (0.055 mL, 0.545 mmol) was added to the vigorously stirred mixture, which was then stirred for 1 hour at room temperature. After removal of NEt_3_ by evaporation, dichloromethane was added and the solution was filtered through a pad (0.5 cm) of silica gel by using ethyl acetate as an eluent. The filtrate was concentrated to about 1 mL volume and chromatographed on a silica gel column (8 × 1 cm). Four fractions were successively eluted with petroleum ether-ethyl acetate (9 : 1), (4 : 1), (3 : 2) mixtures and finally with ethyl acetate. The first yellow-brown fraction gave 3 mg of the initial Cp(CO)_2_FeI. 167 mg (89%) of dibenzylideneacetone was obtained from the second bright yellow fraction. The binuclear FePd complex 4 was isolated in 78% yield (213 mg, 0.415 mmol) as a brown solid after evaporation of the solvent from the third orange fraction. The fourth dark-yellow fraction contained 4 mg (0.014 mmol, 3% yield) of Cp(CO)_2_Fe–C≡C–(2-C_5_H_4_N) (1). A recrystallization of Cp(CO)_2_Fe[μ-C=CH(2-C_5_H_4_N)]PdI from CH_2_Cl_2_–hexane (1 : 2) mixture gave 176 mg of red-brown microcrystals. ^1^H NMR (CD_2_Cl_2_, 25 °C) *δ*, ppm [*J*, Hz]: 5.25 (s, C_5_***H***_5_); 6.91 (d, *J*_HH_ = 8.2, ***H***_*ortho*_ of (2-Pyr)); 7.07 (t, *J*_HH_ = 5.9, ***H***_*meta*_ of (2-Pyr)); 7.54s (=C^2^***H***); 7.69 (t, *J*_HH_ = 7.3, ***H***_*para*_ of (2-Pyr)); 9.41 (d, *J*_HH_ = 3.4, N–C–***H***_*meta*_ of (2-Pyr)).^13^C{^1^H} NMR (CD_2_Cl_2_, 25 °C) *δ*, ppm [*J*, Hz]: 88.3 (s, ***C***_5_H_5_); 115.2 (s, ***C***_*ortho*_ of (2-Pyr)); 121.0 (s, ***C***_*meta*_ of (2-Pyr)); 139.1 (s, ***C***_*para*_ of (2-Pyr)); 140.2 (s, =***C***^2^H); 153.2 (s, N–***C***_*meta*_ of (2-Pyr)); 171.0 (s, ***C***_*ipso*_ of (2-Pyr)); 206.25 (s, Fe–***C***O); 230.77 (s, Fe-***C***O_bridging_); 312.8 (s, μ-***C***^1^). IR (CH_2_Cl_2_*ν*/cm^−1^): 2026s, 1876s (*ν*_CO_), 1600m, 1582m, 1550m, 1466m (*ν*_C=C_ and *ν*_C=N_). IR (KBr *ν*/cm^−1^): 2005s, 1848s (*ν*_CO_), 1599m, 1583m, 1546m, 1464m (*ν*_C=C_ and *ν*_C=N_). Anal. found: C, 32.78%; H, 1.95%; N, 2.73%. Calc. for C_14_H_10_FeINO_2_Pd (513): C, 32.75; H, 1.96%; N, 2.73%.

### Synthesis of Cp(CO)_2_Fe–C≡C–(4-C_6_H_3_N_2_S) (5)

Following the procedure described for preparation of 1–3, complex Cp(CO)_2_Fe–C≡C–(4-C_6_H_3_N_2_S) (5) was obtained from cyclopentadienyliron dicarbonyl iodide (309 mg, 1.016 mmol) and 4-ethynyl-2,1,3-benzothiadiazole (245 mg, 1.531 mmol) with the use of THF (9 mL), DBU (0.23 mL, 234 mg, 1.539 mmol), PdCl_2_(NCMe)_2_ (3 mg, 0.012 mmol), and CuI (10 mg, 0.052 mmol). The product was isolated from the reaction mixture by column chromatography on silica gel (9 × 2 cm). A dark-yellow fraction containing the σ-4-benzothiadiazolylethynyl iron complex was eluted with petroleum ether-ethyl acetate (4 : 1 and 3 : 2) mixture. The complex Cp(CO)_2_Fe–C≡C–(4-C_6_H_3_N_2_S) (5) was obtained as brown-yellow solid after evaporation of the solvent (319 mg, 0.949 mmol, 94%). ^1^H NMR (CD_2_Cl_2_, 25 °C) *δ*, ppm [*J*, Hz]: 5.21 (s, 5H, C_5_***H***_5_); 7.48 (dd, ^3^*J*_HH_ = 7; ^4^*J*_HH_ = 1.5, 1H, ***H***_*ortho*_ of (4-C_6_***H***_3_N_2_S)); 7.50 (dd, ^3^*J*_HH_ = 8.5; ^3^*J*_HH_ = 7.0, ***H***_*meta*_ of (4-C_6_***H***_3_N_2_S)); 7.77 (dd, ^3^*J*_HH_ = 8.4, ^4^*J*_HH_ = 1.5, 1H, ***H***_*para*_ of (4-C_6_***H***_3_N_2_S)). ^13^C{^1^H} NMR (CD_2_Cl_2_, 25 °C) *δ*, ppm [*J*, Hz]: 85.6 (s, ***C***_5_H_5_); 102.9 (s, ≡***C***^2^–); 112.3 (s, –***C***^1^≡); 118.1 (s, ***C***_*para*_ of (4-C_6_H_3_N_2_S)); 121.1 (s, ***C***_*ipso*_ of (4-C_6_H_3_N_2_S)); 129.4 (s, ***C***_*ortho*_ of (4-C_6_H_3_N_2_S)) and 130.1 (s, ***C***_*meta*_ of (4-C_6_H_3_N_2_S)); 154.8 (s, N–***C***_*meta*_ of (4-C_6_H_3_N_2_S)); 155.7 (s, N–***C***_*ortho*_ of (4-C_6_H_3_N_2_S)); 212.3 (s, 2Fe–***C***O). IR (CH_2_Cl_2_, cm^−1^): 2100 (*ν*_C≡C_), 2042, 1997 (*ν*_CO_), 1529 (*ν*_C=C (conj.)_); (KBr, cm^−1^): 2095m (*ν*_C≡C_), 2031s, 1977vs (*ν*_CO_), 1527m (*ν*_C=C (conj.)_). Anal. found: C, 53.46%; H, 2.41%; N, 8.36%. Calc. for C_15_H_8_FeN_2_O_2_S (336): C, 53.60%; H, 2.40%; N, 8.33%.

### X-ray diffraction studies

Crystal data and X-ray experimental details for the complexes 1–3 and 5 are given in Table S4 (the ESI†). Brown-yellow crystals of the complexes suitable for X-ray diffraction analysis were obtained by evaporation of a solution of the complexes in a dichloromethane : hexane mixture = 1 : 2 under argon atmosphere at +5 °C. The experimental data were collected on a Smart Photon II diffractometer (Bruker AXS) with CCD area detector and graphite monochromator using Mo Kα radiation at room temperature. Absorption corrections have been applied using multiscan procedure.^85^ The structures were solved by direct methods and refined by full-matrix least squares on F^2^, using SHELX programs.^86,87^ Hydrogen atoms have been placed in calculated positions and taken into account in the final stages of refinement in the “riding model” approximation.

## Author contributions

Victor V. Verpekin: conceptualization, data curation, funding acquisition, investigation, methodology, supervision, visualization, writing – original draft, writing – review & editing; Oleg V. Semeikin: perform investigation on Pd(0)-catalyzed coupling reactions of Cp(CO)_2_FeI and [(trimethylsilyl)ethynyl]pyridines; Alexander D. Vasiliev: X-ray diffractometry investigation; Alexander A. Kondrasenko: NMR investigation; Yuri A. Belousov: resources; Nikolai A. Ustynyuk: conceptualization, writing – review & editing. All authors have read and agreed to the published version of the manuscript.

## Conflicts of interest

There are no conflicts to declare.

## Supplementary Material

RA-010-D0RA02333G-s001

RA-010-D0RA02333G-s002
