# Supplementary material for: Catalyzed M–C coupling reactions in the synthesis of σ-(pyridylethynyl)dicarbonylcyclopentadienyliron complexes
Source: RSC Adv. 2020 Apr 30;10(29):17014–25. doi: 10.1039/d0ra02333g (PMC9053414; doi:10.1039/d0ra02333g)
Supplement: RA-010-D0RA02333G-s001 [file RA-010-D0RA02333G-s001.pdf]

Electronic Supplementary Material (ESI) for RSC Advances.
This journal is © The Royal Society of Chemistry 2020

Supporting information

Catalyzed M-C coupling reactions in synthesis of σ-(pyridylethynyl)dicarbonylcyclopentadienyliron complexes

Victor V. Verpekin,*, [a] Oleg V. Semeikin,[b] Alexander D. Vasiliev,[c, d] Alexander A. Kondrasenko,[a] Yuri A. Belousov [b] and Nikolai A. Ustynyuk*, [b]

[a] Institute of Chemistry and Chemical Technology SB RAS, Krasnoyarsk Research Center, Siberian Branch of the Russian Academy of Sciences, Akademgorodok 50-24, Krasnoyarsk, 660036, Russian Federation

[b] A. N. Nesmeyanov Institute of Organoelement Compounds, Russian Academy of Sciences, ul. Vavilova 28, Moscow 119991, Russian Federation

[c] Institute of Physics SB RAS, Krasnoyarsk Research Center, Siberian Branch of the Russian Academy of Sciences, Akademgorodok, 50-38, Krasnoyarsk, 660036, Russia

[d] Siberian Federal University, Svobodny Prospect, 79, Krasnoyarsk, 660041, Russia

*Corresponding autors: Victor Verpekin (vvv@sany-ok.ru, vvv@icct.ru), Nikolai A. Ustynyuk (ustynyuk@ineos.ac.ru)

Table of contents

General Information....2
- General procedure for coupling reactions between $Cp(CO)_2FeI$ and [(trimethylsilyl)ethynyl]pyridines..2
- General procedure for coupling reactions between $Cp(CO)_2FeI$ and ethynylpyridines....4
- Synthesis of [Cp(CO)Fe{μ_2-η^1(C_α):η^1(C_α)-κ^1(N)-C_α=C_β(H)(2-C_5H_4N)}(μ-CO)PdI] (**4**)....7
- Synthesis of $Cp(CO)_2Fe$-C≡C-(4-$C_6H_3N_2S$) (**5**). 7

Analytical data....8
- $Cp(CO)_2Fe$-C≡C-(2-C_5H_4N) (**1**)....8
- $Cp(CO)_2Fe$-C≡C-(3-C_5H_4N) (**2**)....8
- $Cp(CO)_2Fe$-C≡C-(4-C_5H_4N) (**3**)....9
- Cp(CO)(PPh_3)Fe-C≡C-(2-C_5H_4N) 9
- [Cp(CO)Fe{μ_2-η^1(C_α):η^1(C_α)-κ^1(N)-C_α=C_β(H)(2-C_5H_4N)}(μ-CO)PdI] (**4**) 9
- $Cp(CO)_2Fe$-C≡C-(4-$C_6H_3N_2S$) (**5**)....9

X-ray diffraction studies 10
- X-ray diffraction study of $Cp(CO)_2Fe$-C≡C-(2-C_5H_4N) (**1**)....10
- X-ray diffraction study of $Cp(CO)_2Fe$-C≡C-(3-C_5H_4N) (**2**)....10
- X-ray diffraction study of $Cp(CO)_2Fe$-C≡C-(4-C_5H_4N) (**3**)....11
- X-ray diffraction study of $Cp(CO)_2Fe$-C≡C-(4-$C_6H_3N_2S$) (**5**)....11

NMR and IR spectra....15
References....19

General Information

All operations and manipulations were carried out under an argon atmosphere. Solvents (dichloromethane, petroleum ether, ethyl acetate, hexane, triethylamine) were purified by distillation from appropriate drying agents and stored under argon. THF was dried by refluxing over sodium/benzophenone and freshly distilled prior to use. The course of reactions was monitored by TLC on Silica gel (Alu foils, Sigma-Aldrich) and IR spectroscopy. Neutral silica gel (Silica 60, 0.2-0.5 mm, Macherey-Nagel) was used for column chromatography. $Pd(Cl)_2(PPh_3)_2$[1], $Pd(Cl)_2(NCMe)_2$[2], $Pd_2(dba)_3{\cdot}CHCl_3$[3], and $Cp(CO)_2FeI$[4] were prepared according to literature procedures. *Ortho*-, *meta*-, *parta*-[(trimethylsilyl)ethynyl]pyridines[5] and 4-(trimethylsilyl)ethynyl-2,1,3-benzothiadiazole[6] were prepared from ethynyltrimethylsilane. *Ortho*- and *para*-ethynylpyridines were synthesised from 2-methyl-4-(n-pyridyl)but-3-yn-2-ol intermediates by elimination of acetone, according to published procedures[7]. *Meta*-ethynylpyridine and 4-ethynyl-2,1,3-benzothiadiazole were obtained by desilylation of *meta*-[(trimethylsilyl)ethynyl]pyridine[8] and 4-(trimethylsilyl)ethynyl-2,1,3-benzothiadiazole[6]. Tetrabutylammonium fluoride solution (1M solution in THF, Aldrich), 1,8-Diazabicyclo[5.4.0]undec-7-ene (Aldrich), 1M and catalyst CuI (“Vekton-M” Ltd.) were purchased and used directly.

Physical-chemical characteristics were obtained in the Krasnoyarsk Regional Centre of Research Equipment, Siberian Branch of the Russian Academy of Sciences. The IR spectra were recorded on the Shimadzu IR Tracer-100 spectrometer (Japan). ^{1}H, $^{13}C\{^{1}H\}$, $^{31}P\{^{1}H\}$, HSQC, and HMBC NMR spectra were obtained using NMR spectrometer AVANCE III 600 (Bruker, Germany). Chemical shifts are reported in ppm units referenced to residual solvent resonances for ^{1}H, $^{13}C\{^{1}H\}$, HSQC, and HMBC spectra or to an external 85% H_3PO_4(aq) standard for $^{31}P\{^{1}H\}$ spectra. The X-ray data for **1- 3** were obtained with the Smart Photon II diffractometer (Bruker AXS, Germany).

General procedure for coupling reactions between $Cp(CO)_2FeI$ and [(trimethylsilyl)ethynyl]pyridines

Cyclopentadienyliron dicarbonyl iodide (1 equiv) and [(trimethylsilyl)ethynyl]pyridine (1.5 equiv) were dissolved in freshly distilled THF, then 1M solution of TBAF (1.5 equiv) in THF was added through syringe. To the resulting vigorously stirred mixture the catalysts (Table 1S) were added. The resulting mixture was stirred and heated (Table 1S). After a time, the reaction mixture was evaporated to dryness; the residue was dissolved in dichloromethane and passed through a pad (0.5 cm) of silica gel by using ethyl acetate as an eluent. The filtrate was concentrated *in vacuo* and chromatographed on silica gel (9×2cm). The column was eluted initially with petroleum ether-dichloromethane mixtures and subsequently with petroleum ether-ethyl acetate mixtures (Table 1S).

Table 1S. The details of the reactions between [(trimethylsilyl)ethynyl]pyridines and cyclopentadienyliron dicarbonyl iodide

entry	Loadings and conditions	
1	$Cp(CO)_2FeI$ (201 mg, 0.661 mmol); TMS-C≡C-(2-C_5H_4N) (174 mg, 0.994 mmol);<br>$PdCl_2(PPh_3)_2$ (45 mg, 0.064 mmol, 10 mol%); CuI (25 mg, 0.131 mmol, 20 mol%);<br>1.00 mL TBAF (1M in THF);THF (8 mL); 40°C; 120 min	
	Eluents	Isolated products, %
	PE:CH_2Cl_2 = 9:1<br>PE:CH_2Cl_2 = 4:1<br>PE:CH_2Cl_2 = 4:1 and 7:3<br>PE:EtOAc = 3:2<br>PE:EtOAc = 1:1<br>PE:EtOAc = 3:7 and EtOAc	$[Cp(CO)_2Fe]_2$ (4 mg, 3%)<br>$Cp(CO)_2FeI$ (61 mg)<br>$Cp(CO)(PPh_3)FeI$ (12 mg, 3%)<br>$[-C{\equiv}C-(2-C_5H_4N)]_2$ (5 mg, 5%);<br>$Cp(CO)(PPh_3)Fe$-C≡C-(2-C_5H_4N) (9 mg, 3%)<br>$Cp(CO)_2Fe$-C≡C-(2-C_5H_4N) (**1**) (106 mg, 58%)
2	Loadings and conditions	
	$Cp(CO)_2FeI$ (205 mg, 0.674 mmol); TMS-C≡C-(2-C_5H_4N) (177 mg, 1.011 mmol);<br>$Pd_2(bda)_3$ (35 mg, 0.034 mmol, 10 mol%); CuI (26 mg, 0.136 mmol, 20 mol%);<br>1.01 mL TBAF (1M in THF); THF (6 mL); 36°C; 90 min	

	Eluents	Isolated products, %
	PE:CH_2Cl_2 = 9:1<br>PE:CH_2Cl_2 = 4:1<br>PE:CH_2Cl_2 = 3:2<br>PE:EtOAc = 3:2<br>PE:EtOAc = 3:7 and EtOAc	[Cp(CO)$_2$Fe]$_2$ (38 mg, 32%);<br>Cp(CO)$_2$FeI (4 mg)<br>dba (14 mg, 58%)<br>[-C≡C-(2-C_5H_4N)]$_2$ (2 mg, 2%);<br>Cp(CO)$_2$Fe-C≡C-(2-C_5H_4N) (**1**) (109 mg, 58%)
3	Loadings and conditions	
	Cp(CO)$_2$FeI (247 mg, 0.813 mmol); TMS-C≡C-(2-C_5H_4N) (212 mg, 1.211 mmol);<br>Pd$_2$(bda)$_3$ (42 mg, 0.041 mmol, 10 mol%); -;<br>1.20 mL TBAF (1M in THF); THF (8 mL); 36°C; 90 min	
	Eluents	Isolated products, %
	PE:CH_2Cl_2 = 9:1<br>PE:CH_2Cl_2 = 4:1<br>PE:CH_2Cl_2 = 3:2<br>PE:EtOAc = 3:2<br>PE:EtOAc = 3:7 and EtOAc	[Cp(CO)$_2$Fe]$_2$ (41mg, 28%);<br>Cp(CO)$_2$FeI (6 mg)<br>dba (25 mg, 86%)<br>[-C≡C-(2-C_5H_4N)]$_2$ (6 mg, 5%);<br>Cp(CO)$_2$Fe-C≡C-(2-C_5H_4N) (**1**) (137mg, 60%)
4	Loadings and conditions	
	Cp(CO)$_2$FeI (150 mg, 0.493 mmol); TMS-C≡C-(2-C_5H_4N) (130 mg, 0.743 mmol);<br>Pd$_2$(bda)$_3$ (5 mg, 0.005 mmol, 2 mol%); -;<br>0.75 mL TBAF (1M in THF); THF (6 mL); 60°C; 90 min	
	Eluents	Isolated products, %
	PE:CH_2Cl_2 = 9:1<br>PE:CH_2Cl_2 = 4:1<br>PE:CH_2Cl_2 = 3:2<br>PE:EtOAc = 3:7 and EtOAc	[Cp(CO)$_2$Fe]$_2$ (32mg, 37%);<br>Cp(CO)$_2$FeI (4 mg)<br>dba (3 mg, 75%)<br>Cp(CO)$_2$Fe-C≡C-(2-C_5H_4N) (**1**) (61 mg, 44%)
5	Loadings and conditions	
	Cp(CO)$_2$FeI (209 mg, 0.688 mmol); TMS-C≡C-(2-C_5H_4N) (180 mg, 1.029 mmol);<br>PdCl$_2$(NCMe)$_2$ (18 mg, 0.069 mmol, 10 mol%); CuI (26 mg, 0.136 mmol, 20 mol%);<br>1.03 mL TBAF (1M in THF); THF (7 mL); 36°C; 90 min	
	Eluents	Isolated products, %
	PE:CH_2Cl_2 = 9:1<br>PE:CH_2Cl_2 = 4:1<br>PE:EtOAc = 3:2<br>PE:EtOAc = 3:7 and EtOAc	[Cp(CO)$_2$Fe]$_2$ (33 mg, 27%);<br>Cp(CO)$_2$FeI (11 mg)<br>[-C≡C-(2-C_5H_4N)]$_2$ (3 mg, 3%);<br>Cp(CO)$_2$Fe-C≡C-(2-C_5H_4N) (**1**) (112 mg, 58%)
6	Loadings and conditions	
	Cp(CO)$_2$FeI (197 mg, 0.648 mmol); TMS-C≡C-(2-C_5H_4N) (170 mg, 0.971 mmol);<br>PdCl$_2$(NCMe)$_2$ (8 mg, 0.031 mmol, 5 mol%); -;<br>0.97 mL TBAF (1M in THF); THF (6 mL); 50°C; 90 min	
	Eluents	Isolated products, %
	PE:CH_2Cl_2 = 9:1<br>PE:CH_2Cl_2 = 4:1 and 7:3<br>PE:EtOAc = 3:7 and EtOAc	[Cp(CO)$_2$Fe]$_2$ (27 mg, 23%);<br>Cp(CO)$_2$FeI (16 mg)<br>Cp(CO)$_2$Fe-C≡C-(2-C_5H_4N) (**1**) (110 mg, 61%)
7	Loadings and conditions	
	Cp(CO)$_2$FeI (278 mg, 0.914 mmol); TMS-C≡C-(4-C_5H_4N) (219 mg, 1.251 mmol);<br>Pd$_2$(bda)$_3$ (47 mg, 0.045 mmol, 10 mol%); CuI (35 mg, 0.183 mmol, 20 mol%);<br>1.25 mL TBAF (1M in THF); THF (10 mL); 36°C; 90 min	
	Eluents	Isolated products, %
	PE:CH_2Cl_2 = 9:1<br>PE:CH_2Cl_2 = 4:1<br>PE:CH_2Cl_2 = 7:3<br>PE:EtOAc = 3:2<br>PE:EtOAc = 1:1 and EtOAc	Cp(CO)$_2$Fe]$_2$ (52 mg, 32%)<br>Cp(CO)$_2$FeI (11 mg)<br>dba (27 mg, 84%)<br>[-C≡C-(4-C_5H_4N)]$_2$ (8 mg, 6%);<br>Cp(CO)$_2$Fe-C≡C-(4-C_5H_4N) (**3**) (124 mg, 49%)
8	Loadings and conditions	
	Cp(CO)$_2$FeI (171 mg, 0.563 mmol); TMS-C≡C-(4-C_5H_4N) (148 mg, 0.846 mmol);<br>Pd$_2$(bda)$_3$ (29 mg, 0.028 mmol, 10 mol%); -;<br>0.85 mL TBAF (1M in THF); THF (6 mL); 36°C; 90 min	
	Eluents	Isolated products, %
	PE:CH_2Cl_2 = 9:1<br>PE:CH_2Cl_2 = 4:1<br>PE:CH_2Cl_2 = 7:3<br>PE:EtOAc = 3:2<br>PE:EtOAc = 1:1 and EtOAc	[Cp(CO)$_2$Fe]$_2$ (34 mg, 34%);<br>Cp(CO)$_2$FeI (4 mg)<br>dba (16 mg, 80%)<br>[-C≡C-(2-C_5H_4N)]$_2$ (4 mg, 5%);<br>Cp(CO)$_2$Fe-C≡C-(4-C_5H_4N) (**3**) (84 mg, 54%)
9	Loadings and conditions	

	$Cp(CO)_2FeI$ (157 mg, 0.516 mmol); TMS-C≡C-(4-C_5H_4N) (136 mg, 0.777 mmol);<br>$PdCl_2(NCMe)_2$ (14 mg, 0.054 mmol, 10 mol%); CuI (20 mg, 0.105 mmol, 20 mol%);<br>0.80 mL TBAF (1M in THF); THF (10 mL); 36°C; 90 min	
	Eluents	Isolated products, %
	PE:CH_2Cl_2 = 9:1<br>PE:CH_2Cl_2 = 4:1<br>PE:EtOAc = 3:2<br>PE:EtOAc = 1:1 and EtOAc	$[Cp(CO)_2Fe]_2$ (27 mg, 30%);<br>$Cp(CO)_2FeI$ (5 mg)<br>[-C≡C-(4-C_5H_4N)]$_2$ (2 mg, 3%);<br>$Cp(CO)_2$Fe-C≡C-(4-C_5H_4N) (**3**) (64 mg, 44%)
10	Loadings and conditions	
	$Cp(CO)_2FeI$ (152 mg, 0.500 mmol); TMS-C≡C-(4-C_5H_4N) (130 mg, 0.743 mmol);<br>$PdCl_2(NCMe)_2$ (7 mg, 0.027 mmol, 5 mol%); -;<br>0.74 mL TBAF (1M in THF); THF (5 mL); 50°C; 90 min	
	Eluents	Isolated products, %
	PE:CH_2Cl_2 = 9:1<br>PE:CH_2Cl_2 = 4:1 and 7:3<br>PE:EtOAc = 1:1 and EtOAc	$[Cp(CO)_2Fe]_2$ (33 mg, 37%)<br>$Cp(CO)_2FeI$ (12 mg)<br>$Cp(CO)_2$Fe-C≡C-(4-C_5H_4N) (**3**) (56 mg, 40%)
11	Loadings and conditions	
	$Cp(CO)_2FeI$ (250 mg, 0.822 mmol); TMS-C≡C-(3-C_5H_4N) (0.234 mL, 216 mg, 1.234 mmol);<br>$Pd_2(bda)_3$ (43 mg, 0.042 mmol, 10 mol%); -;<br>1.23 mL TBAF (1M in THF); THF (10 mL); 36°C; 90 min	
	Eluents	Isolated products, %
	PE:CH_2Cl_2 = 9:1<br>PE:CH_2Cl_2 = 4:1<br>PE:CH_2Cl_2 = 3:2<br>PE:EtOAc = 3:2<br>PE:EtOAc = 3:7 and EtOAc	$Cp(CO)_2Fe]_2$ (39 mg, 27%)<br>$Cp(CO)_2FeI$ (18 mg)<br>dba (21 mg, 72%)<br>[-C≡C-(3-C_5H_4N)]$_2$ (1 mg, 1%);<br>$Cp(CO)_2$Fe-C≡C-(3-C_5H_4N) (**2**) (129 mg, 56%)
12	Loadings and conditions	
	$Cp(CO)_2FeI$ (302 mg, 0.993 mmol); TMS-C≡C-(3-C_5H_4N) (0.280 mL, 258 mg, 1.474 mmol);<br>$PdCl_2(NCMe)_2$ (13 mg, 0.050 mmol, 5 mol%); -;<br>1.50 mL TBAF (1M in THF); THF (12 mL); 50°C; 90 min	
	Eluents	Isolated products, %
	PE:CH_2Cl_2 = 9:1<br>PE:CH_2Cl_2 = 4:1 and 7:3<br>PE:EtOAc = 3:7 and EtOAc	$[Cp(CO)_2Fe]_2$ (64 mg, 36%);<br>$Cp(CO)_2FeI$ (14 mg)<br>$Cp(CO)_2$Fe-C≡C-(3-C_5H_4N) (**2**) (134 mg, 48%)

General procedure for coupling reactions between $Cp(CO)_2FeI$ and ethynylpyridines

Cyclopentadienyliron dicarbonyl iodide (1 equiv) and ethynylpyridine (1.5 equiv) were dissolved in freshly distilled THF (6-15 mL), then DBU (1.5 equiv) and catalysts were added (Table 2S and 3S). The resulting mixture was stirred at room temperature or heated (Table 2S and 3S). After a time, the reaction mixture was evaporated to dryness; the residue was dissolved in dichloromethane and passed through a pad (0.5 cm) of silica gel by using ethyl acetate as an eluent. The filtrate was concentrated *in vacuo* and chromatographed on silica gel (9×2cm). The column was eluted initially with petroleum ether-dichloromethane mixtures and subsequently with petroleum ether-ethyl acetate mixtures (Table 2S and 3S).

Table 2S. The details of Pd(II)/CuI- and Pd(II)-catalyzed coupling reactions of cyclopentadienyliron dicarbonyl iodide and ethynylpyridines

entry	Loadings and conditions	
1	$Cp(CO)_2FeI$ (105 mg, 0.345 mmol); H-C≡C-(2-C_5H_4N) (0.053 mL, 54 mg, 0.524 mmol);<br>$PdCl_2(PPh_3)_2$ (24 mg, 0.034 mmol, 10 mol%); CuI (13 mg, 0.068 mmol, 20 mol%);<br>DBU (0.08 mL, 81 mg, 0.533 mmol);THF (6 mL); 40°C; 30 min	
	Eluents	Isolated products, %
	PE:CH_2Cl_2 = 4:1<br>PE:CH_2Cl_2 = 7:3<br>PE:EtOAc = 1:1<br>PE:EtOAc = 3:7 and EtOAc	$Cp(CO)_2FeI$ (15 mg)<br>$Cp(CO)(PPh_3)FeI$ (6 mg, 3%)<br>$Cp(CO)(PPh_3)$Fe-C≡C-(2-C_5H_4N) (15 mg, 8%)<br>$Cp(CO)_2$Fe-C≡C-(2-C_5H_4N) (**1**) (61 mg, 66%)
2	Loadings and conditions	
	$Cp(CO)_2FeI$ (310 mg, 1.020 mmol); H-C≡C-(2-C_5H_4N) (0.154 mL, 157 mg, 1.524 mmol);<br>$PdCl_2(NCMe)_2$ (26 mg, 0.100 mmol, 10 mol%); CuI (40 mg, 0.209 mmol, 20 mol%);	

	DBU (0.23 mL, 226 mg, 1.487 mmol);THF (10 mL); 24°C; 20 min	
	Eluents	Isolated products, %
	PE:CH_2Cl_2 = 9:1<br>PE:CH_2Cl_2 = 4:1 and 7:3<br>PE:EtOAc = 3:7 and EtOAc	$[Cp(CO)_2Fe]_2$ (9 mg, 5%)<br>$Cp(CO)_2FeI$ (6 mg)<br>$Cp(CO)_2Fe$-C≡C-(2-C_5H_4N) (**1**) (259 mg, 91%)
3	Loadings and conditions	
	$Cp(CO)_2FeI$ (401 mg, 1.319 mmol); H-C≡C-(2-C_5H_4N) (0.200 mL, 204 mg, 1.981 mmol);<br>$PdCl_2(NCMe)_2$ (7 mg, 0.027 mmol, 2 mol%); CuI (51mg, 0.267 mmol, 20 mol%);<br>DBU (0.30 mL, 305 mg, 2.007 mmol);THF (10 mL); 24°C; 20 min	
	Eluents	Isolated products, %
	PE:CH_2Cl_2 = 9:1<br>PE:CH_2Cl_2 = 4:1 and 7:3<br>PE:EtOAc = 3:7 and EtOAc	$[Cp(CO)_2Fe]_2$ (4 mg, 2%)<br>$Cp(CO)_2FeI$ (13 mg)<br>$Cp(CO)_2Fe$-C≡C-(2-C_5H_4N) (**1**) (345 mg, 94%)
4	Loadings and conditions	
	$Cp(CO)_2FeI$ (315 mg, 1.036 mmol); H-C≡C-(2-C_5H_4N) (0.155 mL, 158 mg, 1.534 mmol);<br>$PdCl_2(NCMe)_2$ (3 mg, 0.012 mmol, 1 mol%); CuI (10 mg, 0.052 mmol, 5 mol%);<br>DBU (0.23 mL, 234 mg, 1.539 mmol); THF (10 mL); 60°C; 30 min	
	Eluents	Isolated products, %
	PE:CH_2Cl_2 = 9:1<br>PE:CH_2Cl_2 = 4:1 and 7:3<br>PE:EtOAc = 3:7 and EtOAc	$[Cp(CO)_2Fe]_2$ (7 mg, 4%)<br>$Cp(CO)_2FeI$ (6 mg)<br>$Cp(CO)_2Fe$-C≡C-(2-C_5H_4N) (**1**) (266 mg, 92%)
5	Loadings and conditions	
	$Cp(CO)_2FeI$ (262 mg, 0.862 mmol); H-C≡C-(2-C_5H_4N) (0.132 mL, 135 mg, 1.311 mmol);<br>$PdCl_2(NCMe)_2$ (11 mg, 0.042 mmol, 5 mol%); -;<br>DBU (0.19 mL, 193 mg, 1.270 mmol);THF (10 mL); 60°C; 90 min	
	Eluents	Isolated products, %
	PE:CH_2Cl_2 = 9:1<br>PE:CH_2Cl_2 = 4:1 and 7:3<br>PE:EtOAc = 3:2<br>PE:EtOAc = 3:7 and EtOAc	$[Cp(CO)_2Fe]_2$ (14 mg, 9%)<br>$Cp(CO)_2FeI$ (186 mg)<br>**4** (12 mg, 3% [Fe], 55% [Pd])<br>$Cp(CO)_2Fe$-C≡C-(2-C_5H_4N) (**1**) (35 mg, 15%)
6	Loadings and conditions	
	$Cp(CO)_2FeI$ (249 mg, 0.819 mmol); H-C≡C-(4-C_5H_4N) (130 mg, 1.262 mmol);<br>$PdCl_2(NCMe)_2$ (21 mg, 0.081 mmol, 10 mol%); CuI (32 mg, 0.168 mmol, 20 mol%);<br>DBU (0.19 mL, 193 mg, 1.270 mmol);THF (10 mL); 24°C; 20 min	
	Eluents	Isolated products, %
	PE:CH_2Cl_2 = 7:3<br>PE:EtOAc = 1:1 and EtOAc	$Cp(CO)_2FeI$ (7 mg)<br>$Cp(CO)_2Fe$-C≡C-(4-C_5H_4N) (**3**) (209 mg, 91%)
7	Loadings and conditions	
	$Cp(CO)_2FeI$ (472 mg, 1.553 mmol); H-C≡C-(4-C_5H_4N) (240 mg, 2.330 mmol);<br>$PdCl_2(NCMe)_2$ (8 mg, 0.031 mmol, 2 mol%); CuI (59 mg, 0.309 mmol, 20 mol%);<br>DBU (0.35 mL, 356 mg, 2.342 mmol);THF (15 mL); 24°C; 20 min	
	Eluents	Isolated products, %
	PE:CH_2Cl_2 = 3:2<br>PE:EtOAc = 1:1 and EtOAc	$Cp(CO)_2FeI$ (20 mg)<br>$Cp(CO)_2Fe$-C≡C-(4-C_5H_4N) (**3**) (400 mg, 92%)
8	Loadings and conditions	
	$Cp(CO)_2FeI$ (339 mg, 1.115 mmol); H-C≡C-(4-C_5H_4N) (173 mg, 1.680 mmol);<br>$PdCl_2(NCMe)_2$ (3 mg, 0.012 mmol, 1 mol%); CuI (11 mg, 0.052 mmol, 5 mol%);<br>DBU (0.25 mL, 255 mg, 1.678 mmol);THF (10 mL); 60°C; 30 min	
	Eluents	Isolated products, %
	PE:CH_2Cl_2 = 4:1<br>PE:EtOAc = 3:7 and EtOAc	$Cp(CO)_2FeI$ (3 mg)<br>$Cp(CO)_2Fe$-C≡C-(4-C_5H_4N) (**3**) (299 mg, 96%)
9	Loadings and conditions	
	$Cp(CO)_2FeI$ (310 mg, 1.020 mmol); H-C≡C-(4-C_5H_4N) (156 mg, 1.515 mmol);<br>$PdCl_2(NCMe)_2$ (13 mg, 0.050 mmol, 5 mol%); -;<br>DBU (0.23 mL, 234 mg, 1.539 mmol);THF (8 mL); 60°C; 90 min	
	Eluents	Isolated products, %
	PE:CH_2Cl_2 = 9:1<br>PE:CH_2Cl_2 = 4:1 and 7:3<br>PE:EtOAc = 3:7 and EtOAc	$[Cp(CO)_2Fe]_2$ (13 mg, 7%)<br>$Cp(CO)_2FeI$ (77 mg)<br>$Cp(CO)_2Fe$-C≡C-(4-C_5H_4N) (**3**) (190 mg, 67%)
10	Loadings and conditions	
	$Cp(CO)_2FeI$ (340 mg, 1.118 mmol); H-C≡C-(3-C_5H_4N) (173 mg, 1.681 mmol);<br>$PdCl_2(NCMe)_2$ (29 mg, 0.112 mmol, 10 mol%); CuI (42 mg, 0.220 mmol, 20 mol%);<br>DBU (0.25 mL, 255 mg, 1.678 mmol);THF (10 mL); 24°C; 20 min	

	Eluents	Isolated products, %
	PE:CH_2Cl_2 = 9:1<br>PE:CH_2Cl_2 = 7:3<br>PE:EtOAc = 1:1 and EtOAc	$[Cp(CO)_2Fe]_2$ (26 mg, 13%)<br>$Cp(CO)_2FeI$ (2 mg)<br>$Cp(CO)_2Fe-C\equiv C-(3-C_5H_4N)$ (**2**) (258 mg, 83%)
11	Loadings and conditions	
	$Cp(CO)_2FeI$ (666 mg, 2.191 mmol); $H-C\equiv C-(3-C_5H_4N)$ (330 mg, 3.204 mmol);<br>$PdCl_2(NCMe)_2$ (13 mg, 0.050 mmol, 2 mol%); CuI (84 mg, 0.440 mmol, 20 mol%);<br>DBU (0.48 mL, 488 mg, 3.210 mmol);THF (15 mL); 24°C; 20 min	
	Eluents	Isolated products, %
	PE:CH_2Cl_2 = 4:1<br>PE:EtOAc = 3:7 and EtOAc	$Cp(CO)_2FeI$ (15 mg)<br>$Cp(CO)_2Fe-C\equiv C-(3-C_5H_4N)$ (**2**) (570 mg, 93%)
12	Loadings and conditions	
	$Cp(CO)_2FeI$ (250 mg, 0.822 mmol); $H-C\equiv C-(3-C_5H_4N)$ (127 mg, 1.223 mmol);<br>$PdCl_2(NCMe)_2$ (2 mg, 0.008 mmol, 1 mol%); CuI (8 mg, 0.042 mmol, 5 mol%);<br>DBU (0.18 mL, 183 mg, 1.204 mmol);THF (8 mL); 60°C; 30 min	
	Eluents	Isolated products, %
	PE:CH_2Cl_2 = 4:1<br>PE:EtOAc = 3:7 and EtOAc	$Cp(CO)_2FeI$ (2 mg)<br>$Cp(CO)_2Fe-C\equiv C-(3-C_5H_4N)$ (**2**) (218 mg, 95%)
13	Loadings and conditions	
	$Cp(CO)_2FeI$ (264 mg, 0.868 mmol); $H-C\equiv C-(3-C_5H_4N)$ (134 mg, 1.301 mmol);<br>$PdCl_2(NCMe)_2$ (11 mg, 0.042 mmol, 5 mol%); -;<br>DBU (0.19 mL, 193 mg, 1.270 mmol);THF (8 mL); 60°C; 90 min	
	Eluents	Fractions and eluents
	PE:CH_2Cl_2 = 9:1<br>PE:CH_2Cl_2 = 4:1 and 7:3<br>PE:EtOAc = 1:1 and EtOAc	$[Cp(CO)_2Fe]_2$ (10 mg, 6%)<br>$Cp(CO)_2FeI$ (69 mg)<br>$Cp(CO)_2Fe-C\equiv C-(3-C_5H_4N)$ (**2**) (160 mg, 66%)

Table 3S. The details of Pd(0)/CuI- and Pd(0)-catalyzed coupling reactions of cyclopentadienyliron dicarbonyl iodide and ethynylpyridines

entry	Loadings and conditions	
1	Loadings and conditions	
	$Cp(CO)_2FeI$ (344 mg, 1.132 mmol); $H-C\equiv C-(2-C_5H_4N)$ (0.171 mL, 175 mg, 1.709 mmol);<br>$Pd_2(bda)_3$ (61 mg, 0.059 mmol, 10 mol%); CuI (42 mg, 0.220 mmol, 20 mol%);<br>DBU (0.260 mL, 265 mg, 1.743 mmol);THF (10 mL); 36°C; 90 min	
	Eluents	Isolated products, %
	PE:CH_2Cl_2 = 9:1<br>PE:CH_2Cl_2 = 7:3<br>PE:CH_2Cl_2 = 3:2<br>PE:EtOAc = 3:2<br>PE:EtOAc = 3:7 and EtOAc	$[Cp(CO)_2Fe]_2$ (15 mg, 8%)<br>$Cp(CO)_2FeI$ (14 mg)<br>dba (33 mg, 80%)<br>$[-C\equiv C-(2-C_5H_4N)]_2$ (6 mg, 3%);<br>$Cp(CO)_2Fe-C\equiv C-(2-C_5H_4N)$ (**1**) (267 mg, 84%)
2	Loadings and conditions	
	$Cp(CO)_2FeI$ (404 mg, 1.329 mmol); $H-C\equiv C-(2-C_5H_4N)$ (0.200 mL, 204 mg, 1.981 mmol);<br>$Pd_2(bda)_3$ (67 mg, 0.065 mmol, 10 mol%); -;<br>DBU (0.29 mL, 295 mg, 1.941 mmol);THF (10 mL); 36°C; 90 min	
	Eluents	Isolated products, %
	PE:CH_2Cl_2 = 9:1<br>PE:CH_2Cl_2 = 4:1 and 7:3<br>PE:CH_2Cl_2 = 3:2<br>PE:EtOAc = 1:1<br>PE:EtOAc = 3:7 and EtOAc	$[Cp(CO)_2Fe]_2$ (41 mg, 17%)<br>$Cp(CO)_2FeI$ (7 mg)<br>dba (38 mg, 84%)<br>**4** (48 mg, 7% [Fe], 72% [Pd])<br>$Cp(CO)_2Fe-C\equiv C-(2-C_5H_4N)$ (**1**) (238 mg, 64%)
3	Loadings and conditions	
	$Cp(CO)_2FeI$ (256 mg, 0.842 mmol); $H-C\equiv C-(4-C_5H_4N)$ (130 mg, 1.262 mmol);<br>$Pd_2(bda)_3$ (42 mg, 0.041 mmol, 10 mol%); CuI (32 mg, 0.168 mmol, 20 mol%);<br>DBU (0.188 mL, 191 mg, 1.257 mmol);THF (7 mL); 36°C; 90 min	
	Eluents	Isolated products, %
	PE:CH_2Cl_2 = 9:1<br>PE:CH_2Cl_2 = 7:3<br>PE:CH_2Cl_2 = 3:2<br>PE:EtOAc = 3:2<br>PE:EtOAc = 3:7 and EtOAc	$[Cp(CO)_2Fe]_2$ (9 mg, 6%)<br>$Cp(CO)_2FeI$ (13 mg)<br>dba (21 mg, 72%)<br>$[-C\equiv C-(4-C_5H_4N)]_2$ (2 mg, 2%);<br>$Cp(CO)_2Fe-C\equiv C-(4-C_5H_4N)$ (**3**) (202 mg, 86%)
4	Loadings and conditions	

	$Cp(CO)_2FeI$ (240 mg, 0.789 mmol); H-C≡C-(4-C_5H_4N) (123 mg, 1.194 mmol);<br>$Pd_2(bda)_3$ (43 mg, 0.042 mmol, 10 mol%); -;<br>DBU (0.18 mL, 183 mg, 1.204 mmol);THF (10 mL); 36°C; 90 min	
	Eluents	Isolated products, %
	PE:CH_2Cl_2 = 9:1<br>PE:CH_2Cl_2 = 7:3<br>PE:CH_2Cl_2 = 3:2<br>PE:EtOAc = 3:2<br>PE:EtOAc = 1:1 and EtOAc	$[Cp(CO)_2Fe]_2$ (28 mg, 20%)<br>$Cp(CO)_2FeI$ (9 mg)<br>dba (24 mg, 83%)<br>[-C≡C-(4-C_5H_4N)]$_2$ (5 mg, 4%);<br>$Cp(CO)_2$Fe-C≡C-(4-C_5H_4N) (**3**) (148 mg, 67%)
5	Loadings and conditions	
	$Cp(CO)_2FeI$ (300 mg, 0.987 mmol); H-C≡C-(3-C_5H_4N) (152 mg, 1.476 mmol);<br>$Pd_2(bda)_3$ (48 mg, 0.046 mmol, 10 mol%); CuI (37 mg, 0.194 mmol, 20 mol%);<br>DBU (0.220 mL, 224 mg, 1.474 mmol);THF (12 mL); 36°C; 90 min	
	Eluents	Fractions and eluents
	PE:CH_2Cl_2 = 9:1<br>PE:CH_2Cl_2 = 7:3<br>PE:CH_2Cl_2 = 7:3<br>PE:EtOAc = 3:2<br>PE:EtOAc = 1:1 and EtOAc	$[Cp(CO)_2Fe]_2$ (12 mg, 7%)<br>$Cp(CO)_2FeI$ (12 mg)<br>dba (28 mg, 88%)<br>[-C≡C-(3-C_5H_4N)]$_2$ (4 mg, 3%);<br>$Cp(CO)_2$Fe-C≡C-(3-C_5H_4N) (**2**) (238 mg, 87%)

Synthesis of [Cp(CO)Fe{μ_2-η^1(C_α):η^1(C_α)-κ^1(N)-C_α=C_β(H)(2-C_5H_4N)}(μ-CO)PdI] (4)

Cyclopentadienyliron dicarbonyl iodide (162 mg, 0.533 mmol) and $Pd_2(dba)_3\cdot CHCl_3$ (277 mg, 0.268 mmol) were dissolved in triethylamine (8 mL). 2-ethynylpyridine (0.055 mL, 0.545 mmol) was added to the vigorously stirred mixture, which was then stirred for 1 hour at room temperature. After removal of NEt_3 by evaporation, dichloromethane was added and the solution was filtered through a pad (0.5 cm) of silica gel by using ethyl acetate as an eluent. The filtrate was concentrated to about 1 mL volume and chromatographed on a silica gel column (8 × 1 cm). Four fractions were successively eluted with petroleum ether-ethyl acetate (9:1), (4:1), (3:2) mixtures and finally with ethyl acetate. The first yellow-brown fraction gave 3 mg of the initial $Cp(CO)_2FeI$. 167 mg (89%) of dibenzylideneacetone was obtained from the second bright yellow fraction. The binuclear FePd complex **4** was isolated in 78% yield (213 mg, 0.415 mmol) as a brown solid after evaporation of the solvent from the third orange fraction. The fourth dark-yellow fraction contained 4 mg (0.014 mmol, 3% yield) of $Cp(CO)_2$Fe-C≡C-(2-C_5H_4N) (**1**). A recrystallization of $Cp(CO)_2$Fe(μ-C=CH(2-C_5H_4N)PdI from CH_2Cl_2-hexane (1:2) mixture gave 176 mg of red-brown microcrystals.

Synthesis of $Cp(CO)_2$Fe-C≡C-(4-$C_6H_3N_2S$) (5).

Method 1. Cyclopentadienyliron dicarbonyl iodide (187 mg, 0.615 mmol) and 4-(trimethylsilyl)ethynyl-2,1,3-benzothiadiazole (214 mg, 0.922 mmol) were dissolved in freshly distilled THF, then 0.93 ml of 1M solution of TBAF (0.930 mmol) in THF was added through syringe. To the resulting vigorously stirred mixture $PdCl_2(NCMe)_2$ (16 mg, 0.062 mmol, 10 mol%) was added. The reaction mixture was stirred at 60°C for 90 minutes, and then was evaporated to dryness; the residue was dissolved in dichloromethane and passed through a pad (0.5 cm) of silica gel by using ethyl acetate as an eluent. The filtrate was concentrated *in vacuo* and chromatographed on silica gel (9×2cm). The first yellow-brown fraction eluted with petroleum ether-dichloromethane (9:1) mixture gave 21 mg (0.069 mmol) of the initial $Cp(CO)_2FeI$. 24 mg (0.136 mmol, 22%) of $[Cp(CO)_2Fe]_2$ was obtained from the second red fraction. The third dark-yellow fraction containing the σ-4-benzothiadiazolylethynyl iron complex was eluted with petroleum ether-ethyl acetate (4:1 and 3:2) mixture. The complex $Cp(CO)_2$Fe-C≡C-(4-$C_6H_3N_2S$) (**5**) was obtained as brown-yellow solids after evaporation of the solvent (131 mg, 0.390 mmol, 63%).

Method 2. Cyclopentadienyliron dicarbonyl iodide (1 equiv) and ethynylpyridine (1.5 equiv) were dissolved in freshly distilled THF, then DBU (1.5 equiv) and catalysts were added (Table 4S). The reaction mixture was stirred at 36°C or 60°C for 30 minutes (Table 4S), and then was evaporated to dryness; the

residue was dissolved in dichloromethane and passed through a pad (0.5 cm) of silica gel by using ethyl acetate as an eluent. The filtrate was concentrated *in vacuo* and chromatographed on silica gel (9×2cm). The column was eluted initially with petroleum ether-dichloromethane mixtures and subsequently with petroleum ether-ethyl acetate mixtures (Table 4S).

Table 4S. The details of the reactions between 4-ethynyl-2,1,3-benzothiadiazole and cyclopentadienyliron dicarbonyl iodide

entry	Loadings and conditions	
1	$Cp(CO)_2FeI$ (241 mg, 0.793 mmol); H-C≡C-(4-$C_6H_3N_2S$) (190 mg, 1.188 mmol);<br>$PdCl_2(NCMe)_2$ (10 mg, 0.039 mmol, 5 mol%); CuI (15 mg, 0.079 mmol, 10 mol%);<br>DBU (0.18 mL, 183 mg, 1.204 mmol);THF (7 mL); 36°C; 30 min	
	Eluents	Isolated products, %
	PE:CH_2Cl_2 = 9:1<br>PE:CH_2Cl_2 = 4:1<br>PE:EtOAc = 4:1 and 3:2	$[Cp(CO)_2Fe]_2$ (12mg, 9%)<br>$Cp(CO)_2FeI$ (2 mg)<br>$Cp(CO)_2Fe$-C≡C-(4-$C_6H_3N_2S$) (238 mg, 89%)
2	Loadings and conditions	
	$Cp(CO)_2FeI$ (309 mg, 1.016 mmol); H-C≡C-(4-$C_6H_3N_2S$) (245 mg, 1.531 mmol);<br>$PdCl_2(NCMe)_2$ (3 mg, 0.012 mmol, 1 mol%); CuI (10 mg, 0.052 mmol, 5 mol%);<br>DBU (0.23 mL, 234 mg, 1.539 mmol);THF (9 mL); 60°C; 30 min	
	Eluents	Isolated products, %
	PE:CH_2Cl_2 = 9:1 and 4:1<br>PE:EtOAc = 4:1 and 3:2	$Cp(CO)_2FeI$ (9 mg)<br>$Cp(CO)_2Fe$-C≡C-(4-$C_6H_3N_2S$) (319 mg, 94%)

Analytical data

Cyclopentadienyl iron(II) dicarbonyl dimer $[Cp(CO)_2Fe]_2$, cyclopentadienyliron dicarbonyl iodide $Cp(CO)_2FeI$, Cyclopentadienyliron carbonyl triphenylphosphine iodide $Cp(CO)(PPh_3)FeI$, and dibenzylideneacetone were identified by IR spectroscopy.

$Cp(CO)_2Fe$-C≡C-(2-C_5H_4N) (1)

Anal. Found: C, 60.41%; H, 3.26%; N, 5.01%. Calc. For $C_{14}H_9FeNO_2$ (279): C, 60.25%; H, 3.25%; N, 5.02%.

IR (CH_2Cl_2 ν/ cm^{-1}): 2112s ($\nu_{C\equiv C}$), 2043vs, 1997vs (ν_{CO}), 1582m, 1556w, 1460m ($\nu_{C=C}$ and $\nu_{C=N}$).

IR (KBr ν/ cm^{-1}): 2108s ($\nu_{C\equiv C}$), 2034vs, 1982vs (ν_{CO}), 1582m, 1556w, 1457m ($\nu_{C=C}$ and $\nu_{C=N}$).

^1H NMR (CD_2Cl_2, 25ºC) δ, ppm [*J*, Hz]: 5.14 (s, 5H, $C_5\boldsymbol{H}_5$); 7.02 (ddd, 1H, $^3J_{HH}$ = 7.6 Hz, $^3J_{HH}$ = 5.3 Hz, $^4J_{HH}$ = 0.8 Hz, $\boldsymbol{H}_{para}$ of (2-Pyr) (H^4)); 7.22 (d, 1H, $^3J_{HH}$ = 8.0 Hz, $\boldsymbol{H}_{ortho}$ of (2-Pyr) (H^6)); 7.53 (td, 1H , $^3J_{HH}$ = 7.7 Hz, $^4J_{HH}$ = 1.6 Hz, $\boldsymbol{H}_{meta}$ of (2-Pyr) (H^5)); 8.40 (d, 1H, $^3J_{HH}$ = 5.3 Hz, N-C-$\boldsymbol{H}_{meta}$ of ≡C^2(2-Pyr) (H^3)).

$^{13}C\{^1H\}$ NMR (CD_2Cl_2, 25ºC) δ, ppm [*J*, Hz]: 85.4 (s, $\boldsymbol{C}_5H_5$); 95.4 (s, ≡$\underline{\boldsymbol{C}}^2$-); 116.7 (s, -$\underline{\boldsymbol{C}}^1$≡); 119.9 (s, $\boldsymbol{C}_{meta}$ of (2-Pyr (C^5))); 125.9 (s,N-$\boldsymbol{C}_{meta}$ of (2-Pyr) (C^6)); 135.3 (s, $\boldsymbol{C}_{para}$ of (2-Pyr) (C^4)); 145.8 (s, $\boldsymbol{C}_{ipso}$ of (2-Pyr));149.0 (s, $\boldsymbol{C}_{ortho}$ of (2-Pyr) (C^3)); 212.2 (s, 2Fe-$\boldsymbol{C}$O).

$Cp(CO)_2Fe$-C≡C-(3-C_5H_4N) (2)

Anal. Found: C, 60.21%; H, 3.26%; N, 5.00%. Calc. For $C_{14}H_9FeNO_2$ (279): C, 60.25%; H, 3.25%; N, 5.02%.

IR (CH_2Cl_2 ν/ cm^{-1}): 2111s ($\nu_{C\equiv C}$), 2044vs, 1996vs (ν_{CO}), 1580w, 1561w, 1474w ($\nu_{C=C}$ and $\nu_{C=N}$).

IR (KBr ν/ cm^{-1}): 2103s ($\nu_{C\equiv C}$), 2038vs, 1994vs (ν_{CO}), 1572w, 1555w, 1473w ($\nu_{C=C}$ and $\nu_{C=N}$).

^1H NMR (CD_2Cl_2, 25ºC) δ, ppm [*J*, Hz]: 5.13 (s, 5H, $C_5\boldsymbol{H}_5$); 7.14 (dd, 1H, $^3J_{HH}$ = 7.3 Hz, $^3J_{HH}$ = 5.0 Hz, $\boldsymbol{H}_{meta}$ of (3-Pyr) (H^5)); 7.54 (d, 1H, $^3J_{HH}$ = 8.0 Hz, $\boldsymbol{H}_{para}$ of (3-Pyr) (H^6)); 8.30 (d, 1H, $^3J_{HH}$ = 3.2 Hz, $\boldsymbol{H}_{ortho}$ of (3-Pyr) (H^4)); 8.49 (s, 1H, N-C-$\boldsymbol{H}_{ortho}$ of (3-Pyr) (H^2)).

$^{13}C\{^1H\}$ NMR (CD_2Cl_2, 25ºC) δ, ppm [*J*, Hz]: 85.3 (s, ***C***$_5$H$_5$); 95.7 (s, ≡***C***^{2}-); 112.3 (s, -***C***^{1}≡); 122.4 (s, ***C***$_{meta}$ of (3-Pyr) (C^5)); 124.6 (s, ***C***$_{ipso}$ of (3-Pyr)) 137.3 (s, ***C***$_{para}$ of (3-Pyr) (C^6)); 145.3 (s, ***C***$_{ortho}$ of (3-Pyr) (C^4)); 152.1 (s, N-***C***$_{ortho}$ of (2-Pyr) (C^2)) 212.4 (s, 2Fe-***C***O).

Cp(CO)$_2$Fe-C≡C-(4-C$_5$H$_4$N) (3)

Anal. Found: C, 60.39%; H, 3.24%; N, 5.04%. Calc. For $C_{14}H_9FeNO_2$ (279): C, 60.25%; H, 3.25%; N, 5.02%.

IR (CH_2Cl_2 *v*/ cm^{-1}): 2110s ($\nu_{C\equiv C}$), 2044vs, 1998vs (ν_{CO}), 1591s ($\nu_{C=C}$ and $\nu_{C=N}$).

IR (KBr *v*/ cm^{-1}): 2109s ($\nu_{C\equiv C}$), 2037vs, 1992vs (ν_{CO}), 1589s, 1526w, 1487w ($\nu_{C=C}$ and $\nu_{C=N}$).

^1H NMR (CD_2Cl_2, 25ºC) δ, ppm [*J*, Hz]: 5.13 (s, 5H, C$_5$***H***$_5$); 7.11 (dd, 2H, $^3J_{HH}$ = 5.4 Hz, ***H***$_{meta}$ of (4-Pyr)); 8.39 (d, 2H, $^3J_{HH}$ = 4.7 Hz, ***H***$_{ortho}$ of (4-Pyr)).

$^{13}C\{^1H\}$ NMR (CD_2Cl_2, 25ºC) δ, ppm [*J*, Hz]: 85.6 (s, ***C***$_5$H$_5$); 102.1 (s, ≡***C***^{2}-); 114.2 (s, -***C***^{1}≡); 125.5 (s, ***C***$_{ortho}$ of (4-Pyr); 135.3 (s, ***C***$_{ipso}$ of (4-Pyr)); 149.3 (s, ***C***$_{meta}$ of (3-Pyr)); 212.3 (s, 2Fe-***C***O).

Cp(CO)(PPh$_3$)Fe-C≡C-(2-C$_5$H$_4$N)

Anal. Found: C, 72.74%; H, 4.70%; N, 2.74%. Calc. For $C_{31}H_{24}FeNOP$ (513): C, 72.53%; H, 4.71%; N, 2.73%.

IR (CH_2Cl_2 *v*/ cm^{-1}): 2080m, 1976s (ν_{CO}), 1582s($\nu_{C=C}$ and $\nu_{C=N}$);

^1H NMR ($CDCl_3$, 25ºC) δ, ppm [*J*, Hz]: 4.99s (5H); 6.39br.s (1H); 6.83br s (1H); 7.33-7.42m (9H); 7.62-7.70m (6H); 7.81br.s (1H); 8.32br.s (1H).

$^{13}C\{^1H\}$ NMR ($CDCl_3$, 25ºC) δ, ppm [*J*, Hz]: 81.0; 84.7; 118.8; 120.3; 123.8; 128.2; 129.9; 133.4; 136.1; 135.3; 141.9; 148.5; 219.2.

$^{31}P\{^1H\}$ NMR ($CDCl_3$, 25ºC) δ, ppm [*J*, Hz]: 75.7s.

[Cp(CO)Fe{μ$_2$-η^1(C$_\alpha$):η^1(C$_\alpha$)-κ^1(N)-C$_\alpha$=C$_\beta$(H)(2-C$_5$H$_4$N)}(μ-CO)PdI] (4)

Anal. Found: C, 32.78%; H, 1.95%; N, 2.73%. Calc. For $C_{14}H_{10}FeINO_2Pd$ (513): C, 32.75; H, 1.96%; N, 2.73%.

IR (CH_2Cl_2 *v*/ cm^{-1}): 2026s, 1876s (ν_{CO}), 1600m, 1582m, 1550m, 1466 m ($\nu_{C=C}$ and $\nu_{C=N}$).

IR (KBr *v*/ cm^{-1}): 2005s, 1848s (ν_{CO}), 1599m, 1583m, 1546m, 1464 m ($\nu_{C=C}$ and $\nu_{C=N}$).

^1H NMR (CD_2Cl_2, 25ºC) δ, ppm [*J*, Hz]: 5.25 (s, C$_5$***H***$_5$); 6.91 (d, J_{HH} = 8.2, ***H***$_{ortho}$ of (2-Pyr)); 7.07 (t, J_{HH} = 5.9, ***H***$_{meta}$ of (2-Pyr)); 7.54 s (=C^2***H***); 7.69 (t, J_{HH} = 7.3, ***H***$_{para}$ of (2-Pyr)); 9.41 (d, J_{HH} = 3.4, N-C-***H***$_{meta}$ of (2-Pyr)).

$^{13}C\{^1H\}$ NMR (CD_2Cl_2, 25ºC) δ, ppm [*J*, Hz]: 88.3 (s, ***C***$_5$H$_5$); 115.2 (s, ***C***$_{ortho}$ of (2-Pyr)); 121.0 (s, ***C***$_{meta}$ of (2-Pyr)); 139.1 (s, ***C***$_{para}$ of (2-Pyr)); 140.2 (s, =***C***^{2}H); 153.2 (s, N-***C***$_{meta}$ of (2-Pyr)); 171.0 (s, ***C***$_{ipso}$ of (2-Pyr)); 206.25 (s, Fe-***C***O); 230.77 (s, Fe-***C***O$_{bridging}$); 312.8 (s, μ-***C***^{1}=).

Cp(CO)$_2$Fe-C≡C-(4-C$_6$H$_3$N$_2$S) (5)

Anal. Found: C, 53.46%; H, 2.41%; N, 8.36%. Calc. For $C_{15}H_8FeN_2O_2S$ (336): C, 53.60%; H, 2.40%; N, 8.33%.

IR (CH_2Cl_2, cm^{-1}): 2100 ($\nu_{C\equiv C}$), 2042, 1997 (ν_{CO}), 1529 ($\nu_{C=C\ (conj.)}$); (KBr, cm^{-1}): 2095m ($\nu_{C\equiv C}$), 2031s, 1977vs (ν_{CO}), 1527m ($\nu_{C=C\ (conj.)}$).

^{1}H NMR (CD_2Cl_2, 25ºC) δ, ppm [*J*, Hz]: 5.21 (s, 5H, $C_5\boldsymbol{H}_5$); 7.48 (dd, $^3J_{HH}$ = 7; $^4J_{HH}$ = 1.5, 1H, $\boldsymbol{H}_{ortho}$ of (4-$C_6\boldsymbol{H}_3N_2S$)); 7.50 (dd, $^3J_{HH}$ = 8.5; $^3J_{HH}$ = 7.0, $\boldsymbol{H}_{meta}$ of (4-$C_6\boldsymbol{H}_3N_2S$)); 7.77 (dd, $^3J_{HH}$ = 8.4, $^4J_{HH}$ = 1.5, 1H, $\boldsymbol{H}_{para}$ of (4-$C_6\boldsymbol{H}_3N_2S$)).

$^{13}C\{^1H\}$ NMR (CD_2Cl_2, 25ºC) δ, ppm [*J*, Hz]: 85.6 (s, $\boldsymbol{C}_5H_5$); 102.9 (s, ≡$\boldsymbol{C}^2$-); 112.3 (s, -$\boldsymbol{C}^1$≡); 118.1 (s, $\boldsymbol{C}_{para}$ of (4-$C_6H_3N_2S$)); 121.1 (s, $\boldsymbol{C}_{ipso}$ of (4-$C_6H_3N_2S$)); 129.4 (s, $\boldsymbol{C}_{ortho}$ of (4-$C_6H_3N_2S$)) and 130.1 (s, $\boldsymbol{C}_{meta}$ of (4-$C_6H_3N_2S$)); 154.8 (s, N-$\boldsymbol{C}_{meta}$ of (4-$C_6H_3N_2S$)); 155.7 (s, N-$\boldsymbol{C}_{ortho}$ of (4-$C_6H_3N_2S$)); 212.3 (s, 2Fe-$\boldsymbol{C}$O).

X-ray diffraction studies

X-ray diffraction study of Cp(CO)$_2$Fe-C≡C-(2-C_5H_4N) (1)

Brown-yellow crystals of the dicarbonyl(2-pyridylethynyl)(η^5-cyclopentadienyl)iron suitable for X-ray diffraction analysis were obtained by evaporation of a solution of the complex in a dichloromethane : hexane mixture = 1:2 under argon atmosphere at +5°C. $C_{14}H_9FeNO_2$, monoclinic, *P 2₁/n*, a = 9.4756(2), b = 9.7388(2), c = 13.6391(3) Å, β = 108.8340(10), V = 1191.24(4) Å^3, Z = 4. The experimental data were collected using fragment of a crystal with dimensions of 0.56 ×0.41 ×0.40 mm on a Smart Photon II diffractometer (Bruker AXS, CCD area detector, graphite monochromator, MoKα radiation, λ = 0.71073Å, 2θ ≤ 60°); 16740 reflections were obtained at 296 K, 3475 were unique. The experimental completeness was 98.8%. Absorption corrections (μ_{Mo} = 1.256 mm^{-1}) have been applied using multiscan procedure[9], R_{int} = 0.0312. The structure was solved by direct methods and refined by full-matrix least squares on F^2, using SHELX programs [10,11]. Hydrogen atoms have been placed in calculated positions and taken into account in the final stages of refinement in the "riding model" approximation. Refinement converged at a final R1 = 0.0310 for reflections with $I_o > 2\sigma_I$ and 0.044 for all data; wR2 = 0.082, GooF = 1.049, 163 refined parameters. The supplementary crystallographic data for compound **1** have been deposited with the Cambridge Crystallographic Data Centre, CCDC No. 1973678.

X-ray diffraction study of Cp(CO)$_2$Fe-C≡C-(3-C_5H_4N) (2)

Brown-yellow crystals of the dicarbonyl(3-pyridylethynyl)(η^5-cyclopentadienyl)iron suitable for X-ray diffraction analysis were obtained by evaporation of a solution of the complex in a dichloromethane : hexane mixture = 1:2 under argon atmosphere at +5°C. $C_{14}H_9FeNO_2$, monoclinic, *P 2₁/n*, a = 9.4258(5), b = 9.5785(5), c = 13.8330(7) Å, β = 109.5340(10), V = 1177.03(11) Å^3, Z = 4. The experimental data were collected using fragment of a crystal with dimensions of 0.51 ×0.42 ×0.36 mm on a Smart Photon II diffractometer (Bruker AXS, CCD area detector, graphite monochromator, MoKα radiation, λ = 0.71073Å, 2θ ≤ 58°); 15154 reflections were obtained at 296 K, 3115 were unique. The experimental completeness was 100%. Absorption corrections (μ_{Mo} = 1.271 mm^{-1}) have been applied using multiscan procedure[9], R_{int} = 0.0654. The structure was solved by direct methods and refined by full-matrix least squares on F^2, using SHELX programs [10,11]. Hydrogen atoms have been placed in calculated positions and taken into account in the final stages of refinement in the "riding model" approximation. Refinement converged at a final R1 = 0.0395 for reflections with $I_o > 2\sigma_I$ and 0.061 for all data; wR2 = 0.09, GooF = 1.046, 163 refined parameters. The supplementary crystallographic data for compound **2** have been deposited with the Cambridge Crystallographic Data Centre, CCDC No. 1973917.

X-ray diffraction study of Cp(CO)$_2$Fe-C≡C-(4-C_5H_4N) (3)

Brown-yellow crystals of the dicarbonyl(4-pyridylethynyl)(η^5-cyclopentadienyl)iron suitable for X-ray diffraction analysis were obtained by evaporation of a solution of the complex in a dichloromethane : hexane mixture = 1:2 under argon atmosphere at +5°C. $C_{14}H_9FeNO_2$, monoclinic, *C 2/c*, a = 16.9444(13), b = 9.1089(7), c = 17.4069(14) Å, β = 114.1839(17) V = 2450.9(3) Å^3, Z = 8. The experimental data were collected using fragment of a crystal with dimensions of 0.61 ×0.32 ×0.08 mm on a Smart Photon II diffractometer (Bruker AXS, CCD area detector, graphite monochromator, MoKα radiation, λ = 0.71073Å, 2θ ≤ 54°); 13414 reflections were obtained at 296 K, 2678 were unique. The experimental completeness was 99.8%.Absorption corrections (μ_{Mo} = 1.221 mm^{-1}) have been applied using multiscan procedure[9], R_{int} = 0.0445. The structure was solved by direct methods and refined by full-matrix least squares on F^2, using SHELX programs [10,11]. Hydrogen atoms have been placed in calculated positions and taken into account in the final stages of refinement in the “riding model” approximation. Refinement converged at a final R1 = 0.0530 for reflections with $I_o>2\sigma_I$ and 0.0718 for all data; wR2 = 0.1245, GooF = 1.095, 163 refined parameters. The supplementary crystallographic data for the compound **3** have been deposited with the Cambridge Crystallographic Data Centre, CCDC No. 1973260. The data can be obtained free of charge via http://www.ccdc.cam.ac.uk or e-mail: deposit@ccdc.cam.ac.uk.

X-ray diffraction study of Cp(CO)$_2$Fe-C≡C-(4-$C_6H_3N_2S$) (5)

Brown-yellow crystals of the dicarbonyl(4-ethynyl-2,1,3-benzothiadiazole)(η^5-cyclopentadienyl)iron suitable for X-ray diffraction analysis were obtained by evaporation of a solution of the complex in a dichloromethane : hexane mixture = 1:2 under argon atmosphere at +5°C. $C_{15}H_8FeN_2O_2S$, monoclinic, *P* 2*1/n*, a = 6.4302(5), b = 11.8451(10), c = 17.9823(15) Å, β = 93.765(2), V = 1366.69(19) Å^3, Z = 4. The experimental data were collected using fragment of a crystal with dimensions of 0.14 × 0.31 × 0.44 mm on a Smart Photon II diffractometer (Bruker AXS, CCD area detector, graphite monochromator, MoKα radiation, λ = 0.71073 Å, 2θ ≤ 54°); 14867 reflections were obtained at 296 K, 2964 were unique. The experimental completeness was 100%. Absorption corrections (μ_{Mo} = 1.260 mm^{-1}) have been applied using multiscan procedure[9], R_{int} = 0.0686. The structure was solved by direct methods and refined by full-matrix least squares on F^2, using SHELX programs [10,11]. Hydrogen atoms have been placed in calculated positions and taken into account in the final stages of refinement in the “riding model” approximation. Refinement converged at a final R1 = 0.0496 for reflections with $I_o>2\sigma_I$ and 0.0738 for all data; wR2 = 0.1076, GooF = 1.076, 190 refined parameters. The supplementary crystallographic data for compound **5** have been deposited with the Cambridge Crystallographic Data Centre, CCDC No. 1982510.

Table 5S. Crystal data and X-ray experimental details for complexes **1-3** and **5**.

Complex	**1**	**2**	**3**	**5**
Empirical formula	$C_{14}H_9FeNO_2$	$C_{14}H_9FeNO_2$	$C_{14}H_9FeNO_2$	$C_{15}H_8FeN_2O_2S$
Formula weight	279.07			336.14
Temperature/K	296			
Crystal system	monoclinic			
Space group	*P*2$_1$/*n*	*P*2$_1$/*n*	*C*2/*c*	*P*2$_1$/*n*
a/Å	9.4756(2)	9.4258(5)	16.9444(13)	6.4302(5)
b/Å	9.7388(2)	9.5785(5)	9.1089(7)	11.8451(10)
c/Å	13.6391(3)	13.8330(7)	17.4069(14)	17.9823(15)
β/°	108.8340(10)	109.5340(10)	114.1838(17)	93.765(2)
Volume/Å^3	1191.24(4)	1177.03(11)	2450.9(3)	1366.69(19)
Z	4	4	8	4
d_{calc}/(g·cm^{-3})	1.556	1.575	1.513	1.634
μ/mm^{-1}	1.256	1.271	1.221	1.260
F(000)	568	568	1136.0	680
Crystal size/mm^3	0.40 × 0.41 × 0.56	0.51 ×0.42 ×0.36	0.08 × 0.32 × 0.61	0.14 × 0.31 × 0.44
Radiation	MoKα (λ = 0.71073)			
2Θ range for data collection/°	4.62 to 60.00	4.61 to 58.00	5.13 to 54.00	4.12 to 54.00
Index ranges	-13 ≤ h ≤ 13,<br>-13 ≤ k ≤ 13,<br>-19 ≤ l ≤ 19	-12 ≤ h ≤ 12,<br>-13 ≤ k ≤ 13,<br>-18 ≤ l ≤ 18	-21 ≤ h ≤ 21,<br>-11 ≤ k ≤ 11,<br>-22 ≤ l ≤ 22	-8 ≤ h ≤ 8,<br>-15 ≤ k ≤ 15,<br>-22 ≤ l ≤ 22
Reflections collected	16740	15154	13414	14867
Uniq. refl./R(int)/R(sigma)	3475/0.0312/0.0228	3115/0.0654/0.0470	2678/0.0445/0.0300	2964/0.0686/0.0467
parameters/restraints	163/0	163/0	163/0	190/0
Goodness-of-fit on F^2	1.049	1.046	1.095	1.076
Final R_1 [I>=2σ (I)]	0.0310	0.0395	0.0530	0.0496
Final R_1, wR_2 [all data]	0.044, 0.082	0.061, 0.090	0.0718, 0.1245	0.0738, 0.1076
$\Delta\rho_{min}/\Delta\rho_{max}$ (e/Å^3)	-0.27/0.26	-0.35/0.30	-0.28/0.40	-0.39/0.31

Table 6S. X-ray crystallographic data for the $Cp(CO)_2Fe-C\equiv C-(n-C_5H_4N)$ [n = *ortho* (**1**), *meta* (**2**), *para* (**3**)] and $Cp(CO)_2Fe-C\equiv C-(4-C_6H_3N_2S)$ (**5**)

Complexes	Selected interatomic distances (Å) and bond angles (◦)
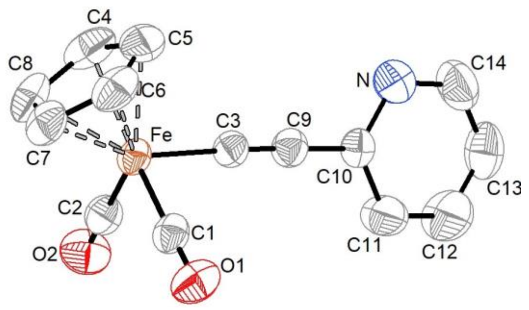<br>$Cp(CO)_2Fe-C\equiv C-(2-C_5H_4N)$ (**1**)	*distances (Å):* Fe-C3 = 1.9159(17), C3-C9 = 1.204(2), C9-C10 = 1.436(2), C10-C11 = 1.366(3), C11-C12 = 1.357(3), C12-C13 = 1.344(4), C14-N = 1,367(3), N-C10 = 1.349(3), C14-C13 = 1.352(4), Fe-C1 = 1.7686(19), C1-O1 = 1.133(2), Fe-C2 = 1.7721(19), C2-O2 = 1.133(2), Fe-Cp = 1.7223(8);<br>*bond angles (°)*: Fe-C3-C9 = 175.94(16), C3-C9-C10 = 176.54(19), Fe-C1-O1 = 178.29(17), Fe-C2-O2 = 178.38(18), C10-C11-C12 = 119.3(2), C11-C12-C13 = 121.1(2), C11-C10-N = 121.57(17), C10-N-C14 = 116.6(2), N-C14-C13 = 123.6(2), C9-C10-C11 = 119.14(17), N-C10-C9 = 119.29(17), C1-Fe-C2 = 94.43(8), C3-Fe-C1 = 89.26(8), C3-Fe-C2 = 87.11(8);<br>*torsion angles (°)*: C1-Fe-C13-C12 = 45.06(2);<br>*angles between planes (°)*: (C4-C5-C6-C7-C8):(C10-C11-C12-C13-N-C14) = 84.51(7), (O1-C1-Fe-C2-O2):(C10-C11-C12-C13-N-C14) = 81.38(6), (C10-C11-C12-C13-N-C14):(Fe-C3-C9-C10-C11) = 5.9(2)
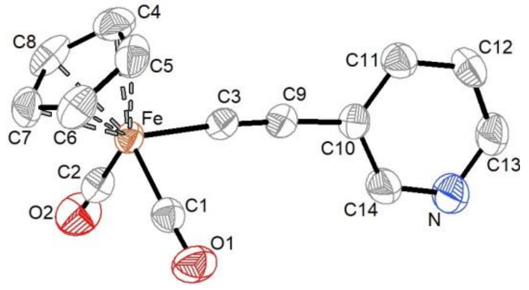<br>$Cp(CO)_2Fe-C\equiv C-(3-C_5H_4N)$ (**2**)	*distances (Å):* Fe-C3 = 1.919(2), C3-C9 = 1.200(3), C9-C10 = 1.437(3), C10-C11 = 1.389(3), C11-C12 = 1.381(4), C12-C13 = 1.368(4), C13-N = 1,326(4), N-C14 = 1.326(4), C14-C10 = 1.392(4), Fe-C1 = 1.763(3), C1-O1 = 1.137(3), Fe-C2 = 1.769(3), C2-O2 = 1.136(3), Fe-Cp = 1.720;<br>*bond angles (°)*: Fe-C3-C9 = 174.3(2), C3-C9-C10 = 176.8(3), Fe-C1-O1 = 178.6(2), Fe-C2-O2 = 178.0(2), C10-C11-C12 = 119.4(2), C11-C12-C13 = 119.2(3), C12-C13-N = 123.3(3), C13-N-C14 = 116.8(3), N-C14-C10 = 125.4(3), C14-C10-C11 = 115.9(2), C14-C10-C9 = 120.1(2), C1-Fe-C2 = 94.9(1), C3-Fe-C1 =86.8(1), C3-Fe-C2 = 89.4(1);<br>*torsion angles (°)*: C1-Fe-C13-N = 49.70(2);<br>*angles between planes (°)*: (C4-C5-C6-C7-C8):(C10-C11-C12-C13-N-C14) = 84.58(8), (O1-C1-Fe-C2-O2):(C10-C11-C12-C13-N-C14) = 80.97(7), (C10-C11-C12-C13-N-C14):(Fe-C3-C9-C10-C11) = 6.4(3)

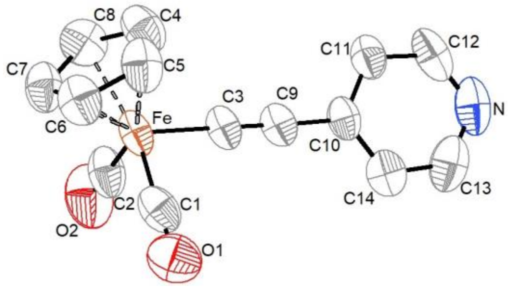<br>Cp(CO)$_2$Fe-C≡C-(4-C$_5$H$_4$N) (**3**)	distances (Å): Fe-C3 = 1.906(3), C3-C9 = 1.203(5), C9-C10 = 1.423(4), C10-C11 = 1.386(5), C11-C12 = 1.386(5), C12-N = 1.313(6), N-C13 = 1,313(6), C13-C14 = 1.375(5), C14-C10 = 1.384(5), Fe-C1 = 1.766(5), C1-O1 = 1.132(5), Fe-C2 = 1.762(4), C2-O2 = 1.123(4), Fe-Cp = 1.719;<br>bond angles (°): Fe-C3-C9 = 179.4(4), C3-C9-C10 = 177.7(5), Fe-C1-O1 = 179.8(4), Fe-C2-O2 = 178.5(4), C9-C10-C11 = 121.8(4), C10-C11-C12 = 119.0(4), C11-C12-N = 124.8(4), C12-N-C13 = 115.7(4), N-C13-C14 = 124.8(5), C13-C14-C10 = 119.6(4), C14-C10-C9 = 122.1(3), C14-C10-C11 = 116.1(3), C1-Fe-C2 = 95.02(19), C3-Fe-C1 =89.68(17), C3-Fe-C2 = 89.07(16);<br>*torsion angles (°)*: C1-Fe-N-C13 = 21.124(4);<br>*angles between planes (°)*: (C4-C5-C6-C7-C8):(C10-C11-C12-N-C13-C14) = 68.64(14), (O1-C1-Fe-C2-O2):(C10-C11-C12-N-C13-C14) = 88.27(8), (C10-C11-C12-N-C13-C14):(Fe-C3-C9-C10-C11) = 0.6(3)
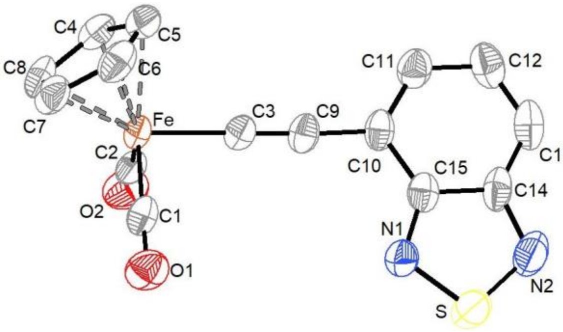<br>Cp(CO)$_2$Fe-C≡C-(4-C$_6$H$_3$N$_2$S) (**5**)	distances (Å): Fe-C3 = 1.917(3), C3-C9 = 1.208(4), C9-C10 = 1.435(4), C10-C11 = 1.380(5), C11-C12 = 1.416(5), C12-C13 = 1.360(6), C13-C14 = 1,399(5), C14-C15 = 1.430(5), C15-C10 = 1.426(5), C14-N2 = 1.358(5), C15-N1 = 1.347(4), N1-S = 1.609(3), N2-S = 1.598(4), Fe-C1 = 1.761(4), C1-O1 = 1.144(4), Fe-C2 = 1.764(4), C2-O2 = 1.145(4), Fe-Cp = 1.7222(16);<br>bond angles (°): Fe-C3-C9 = 178.6(3), C3-C9-C10 = 174.6(4), Fe-C1-O1 = 178.8(3), Fe-C2-O2 = 177.9(3), C9-C10-C11 = 123.2(3), C10-C11-C12 = 124.1(4), C11-C12-C13 = 120.1(4), C12-C13-C14 = 118.8(3), C13-C14-C15 = 120.9(3), C14-C15-C10 = 120.5(3), C15-C10-C9 = 121.3(3), C15-C10-C11 = 115.5(3), C15-C14-N2 = 112.4(4), C14-N2-S = 106.6(3), N2-S-N1 = 101.4(2), S-N1-C15 = 106.1(2), N1-C15-C14 = 113.4(3), C1-Fe-C2 = 95.16(15), C3-Fe-C1 =88.80(15), C3-Fe-C2 = 89.56(14);<br>*torsion angles (°)*: C1-Fe-C13-C14 = 12.7(4);<br>*angles between planes (°)*: (C4-C5-C6-C7-C8):(C10-C11-C12-C13-C14-C15) = 71.22(12), (O1-C1-Fe-C2-O2):(C10-C11-C13-C12-C14-C15) = 81.40(8), (C10-C11-C13-C12-C14-C15):(Fe-C3-C9-C10-C11) = 8.6(3)

NMR and IR spectra

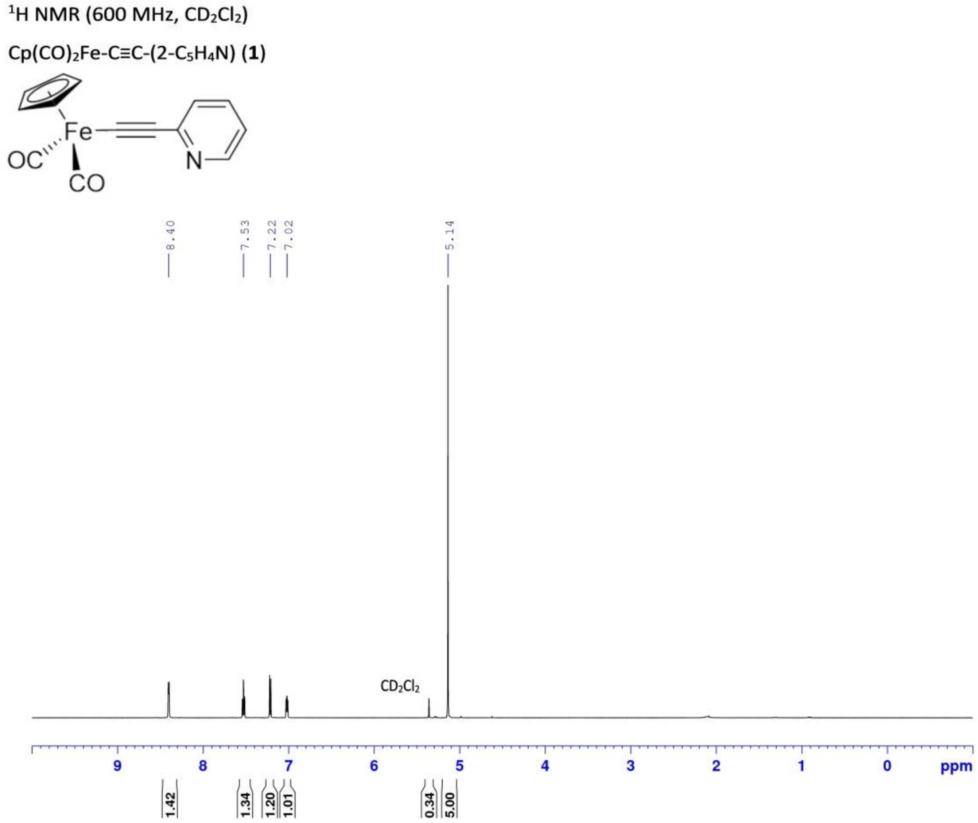

Figure 1S. ^{1}H NMR spectrum of $Cp(CO)_2Fe-C\equiv C-(2-C_5H_4N)$ (**1**) (600 MHz, CD_2Cl_2)

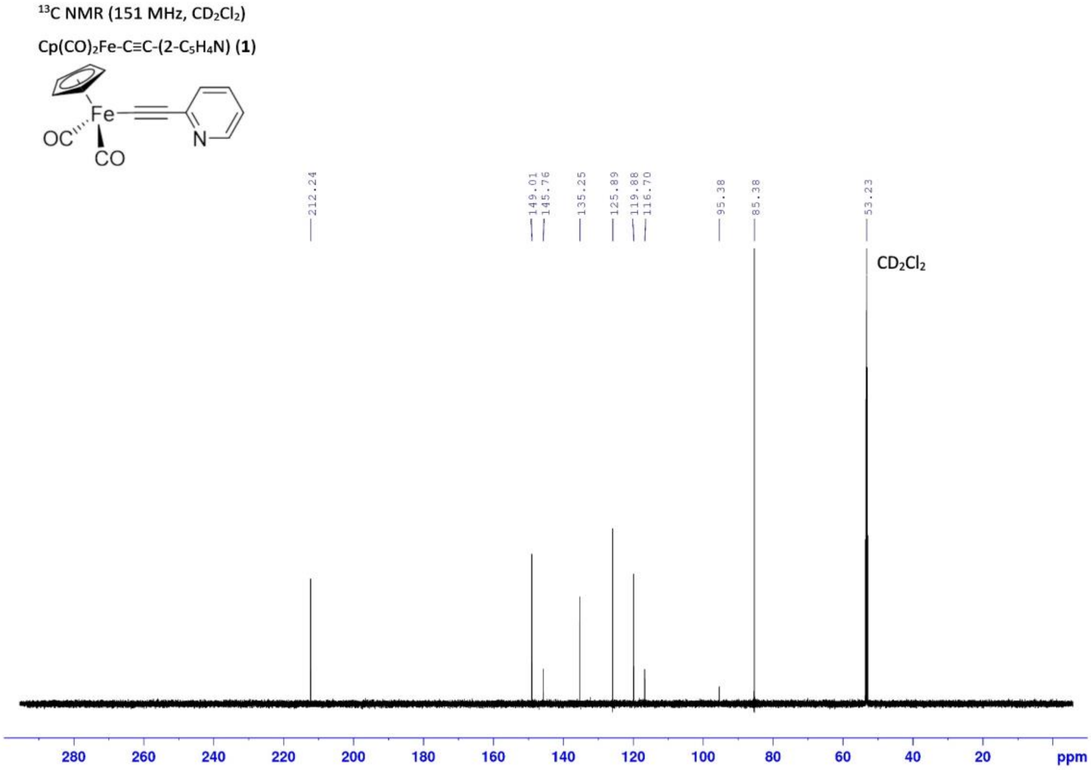

Figure 2S. ^{13}C NMR spectrum of $Cp(CO)_2Fe-C\equiv C-(2-C_5H_4N)$ (**1**) (151 MHz, CD_2Cl_2)

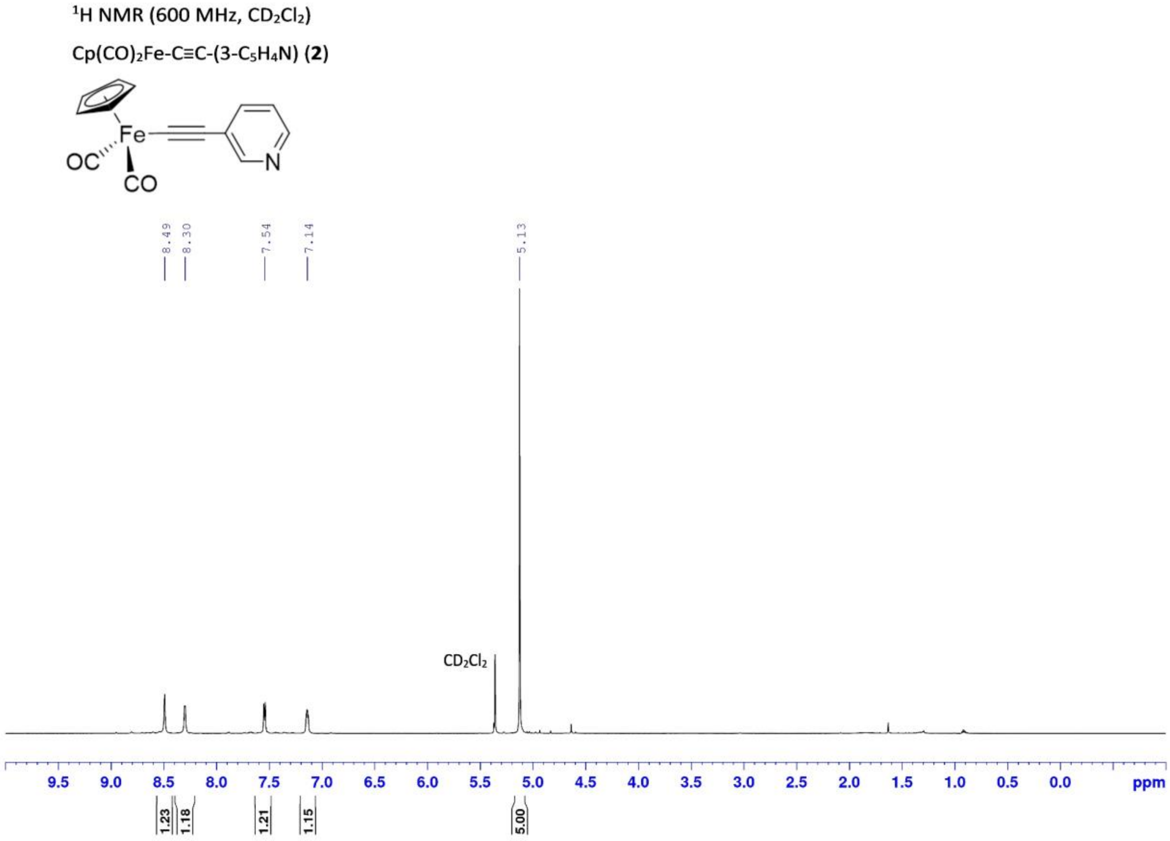

Figure 3S. ^{1}H NMR spectrum of $Cp(CO)_2Fe-C\equiv C-(3-C_5H_4N)$ (**2**) (600 MHz, CD_2Cl_2)

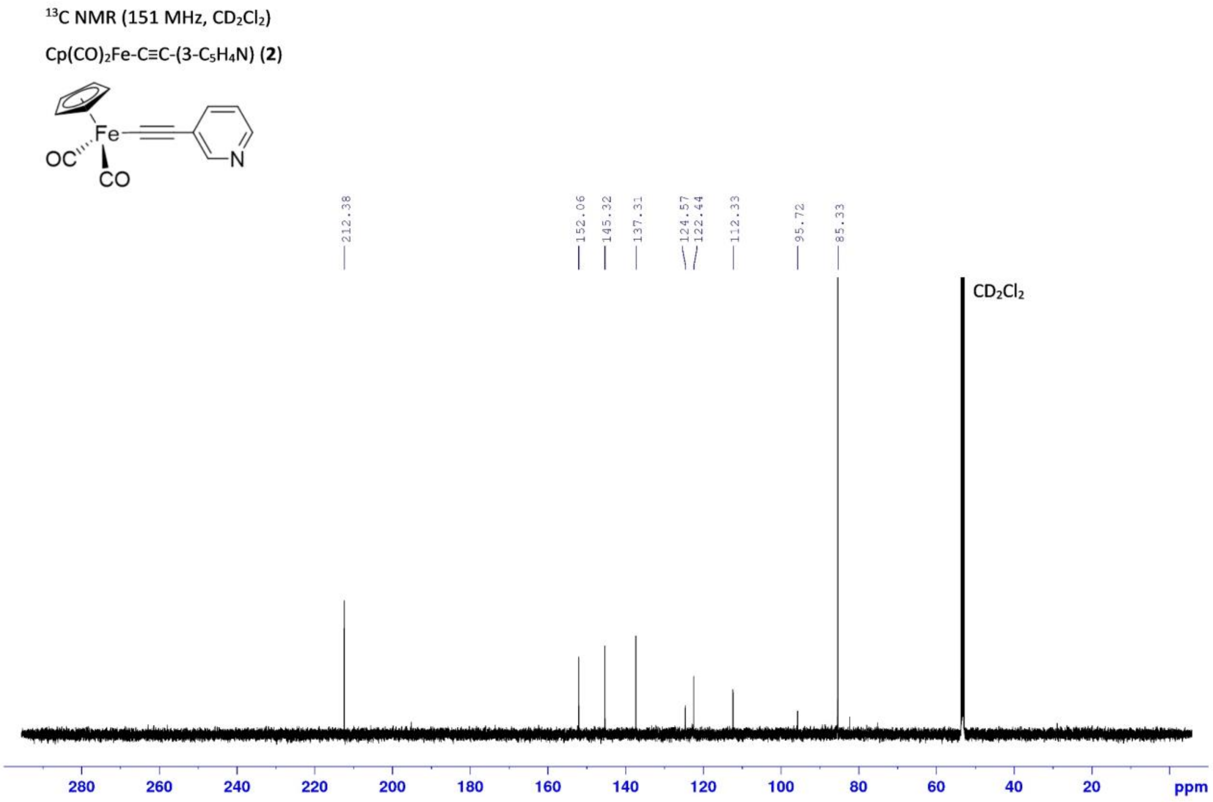

Figure 4S. ^{13}C NMR spectrum of $Cp(CO)_2Fe-C\equiv C-(3-C_5H_4N)$ (**2**) (151 MHz, CD_2Cl_2)

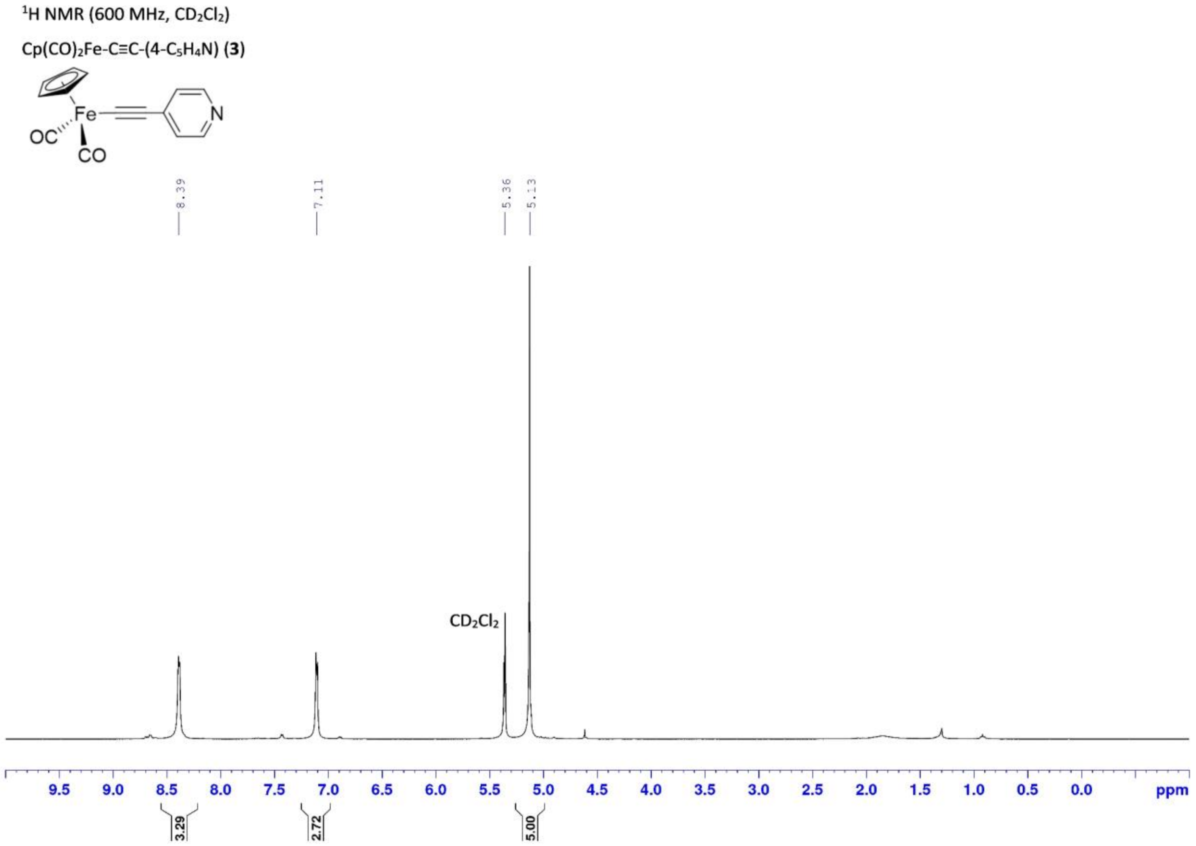

Figure 5S. ^{1}H NMR spectrum of $Cp(CO)_2Fe$-C≡C-(4-C_5H_4N) (**3**) (600 MHz, CD_2Cl_2)

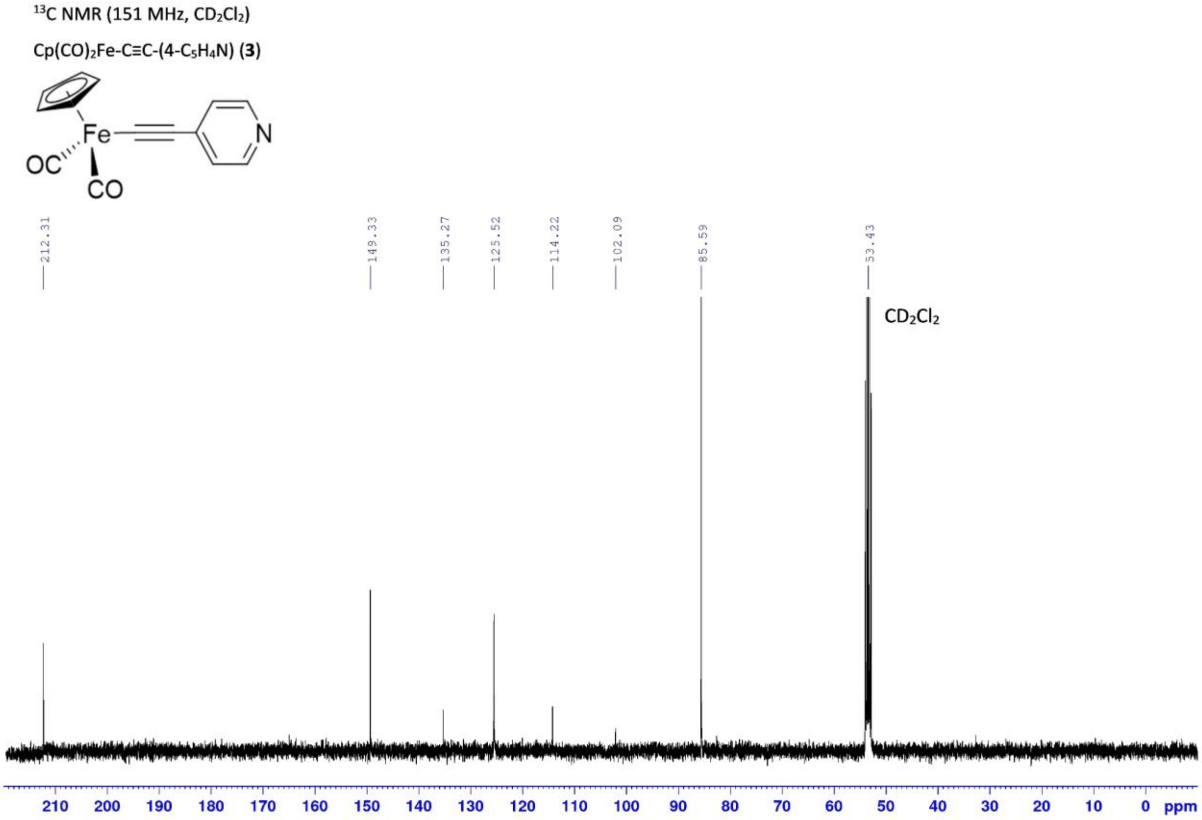

Figure 6S. ^{13}C NMR spectrum of $Cp(CO)_2Fe$-C≡C-(4-C_5H_4N) (**3**) (151 MHz, CD_2Cl_2)

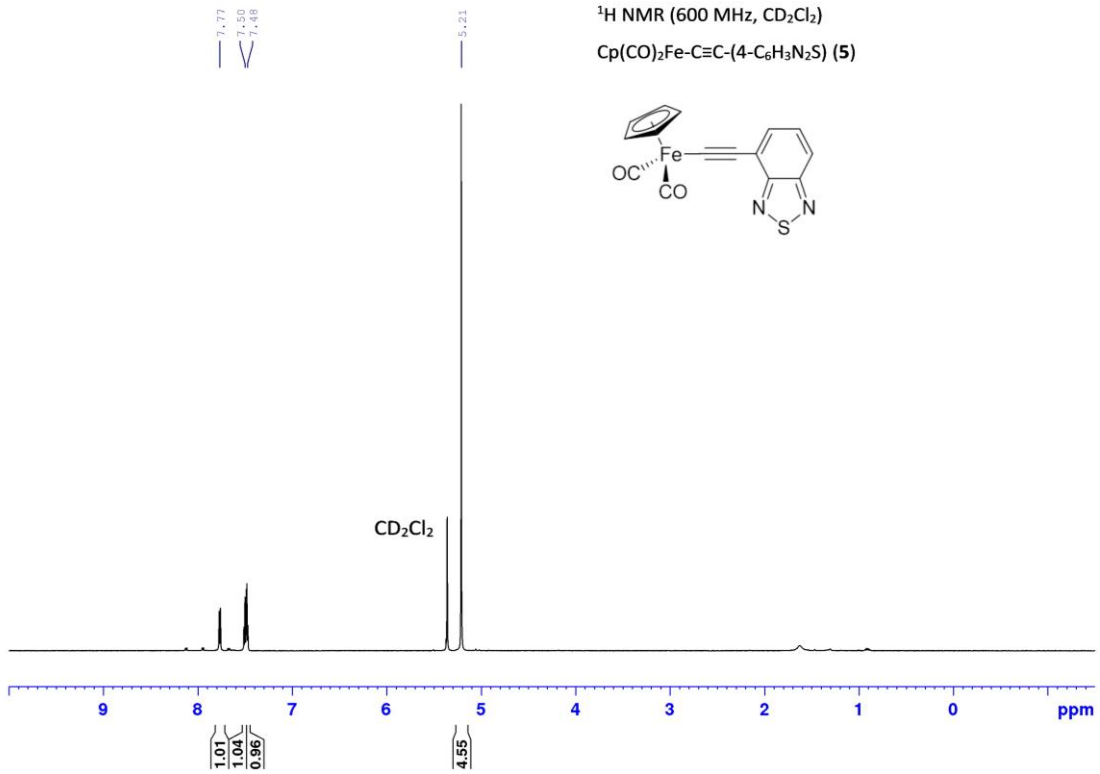

Figure 7S. ^{1}H NMR spectrum of Cp(CO)$_2$Fe-C≡C-(4-C$_6$H$_3$N$_2$S) (**5**) (600 MHz, CD$_2$Cl$_2$)

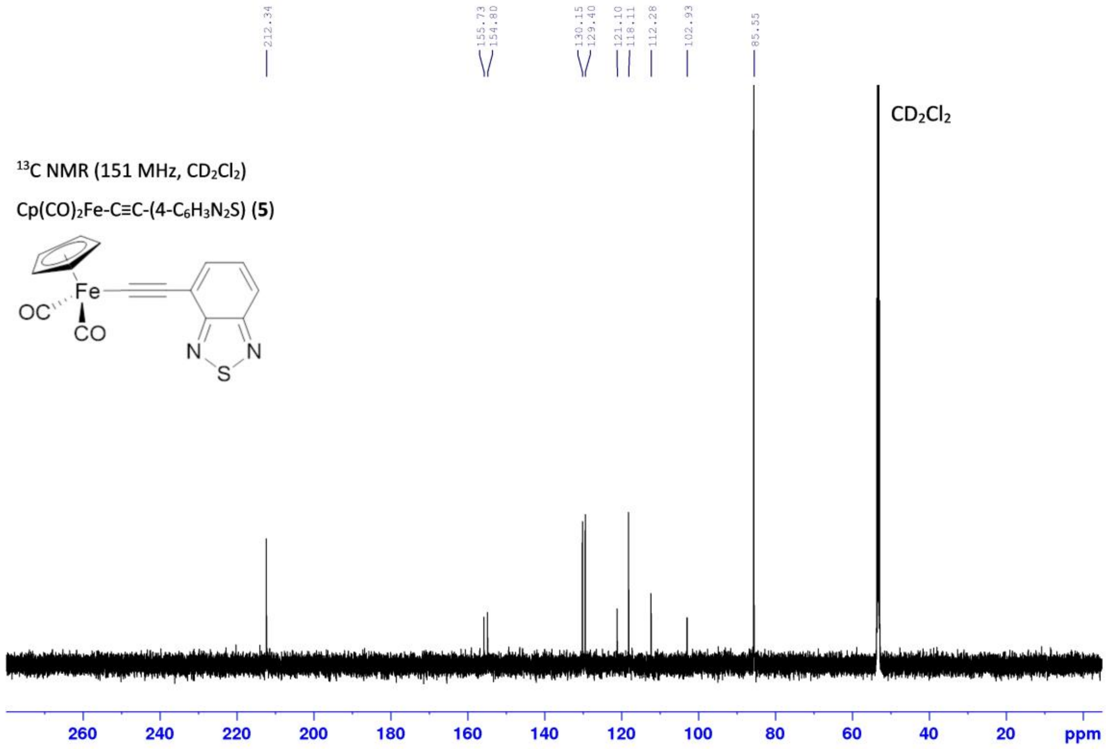

Figure 8S. ^{13}C NMR spectrum of Cp(CO)$_2$Fe-C≡C-(4-C$_6$H$_3$N$_2$S) (**5**) (151 MHz, CD$_2$Cl$_2$)

References

(1) Herrmann, W. A. *Synthetic Methods of Organometallic and Inorganic Chemistry. Vol. 1*; Herrmann, W., Salzer, A., Eds.; G. Thieme: Stuttgart, Germany., 1996.

(2) Anderson, G. K.; Lin, M.; Sen, A.; Gretz, E. Bis(Benzonitrile)Dichloro Complexes of Palladium and Platinum. In *Inorganic synthesis, Vol.28*; Angelici, R. J., Ed.; 1990; pp 60–63. https://doi.org/10.1002/9780470132593.ch13.

(3) Zalesskiy, S. S.; Ananikov, V. P. Pd2(Dba)3 as a Precursor of Soluble Metal Complexes and Nanoparticles: Determination of Palladium Active Species for Catalysis and Synthesis. *Organometallics* **2012**, *31* (6), 2302–2309. https://doi.org/10.1021/om201217r.

(4) King, R. B.; Stone, F. G. A.; Jolly, W. L.; Austin, G.; Covey, W.; Rabinovich, D.; Steinberg, H.; Tsugawa, R. Cyclopentadienyl Metal Carbonyls and Some Derivatives. In *Inorganic Syntheses, Volume 7*; Jacob Kleinberg, Ed.; John Wiley & Sons, Ltd, 1963; pp 99–115. https://doi.org/10.1002/9780470132388.ch31.

(5) Sakamoto, T.; Shiraiwa, M.; Kondo, Y.; Yamanaka, H. A Facile Synthesis of Ethynyl-Substituted Six-Membered N -Heteroaromatic Compounds. *Synthesis (Stuttg).* **1983**, *1983* (04), 312–314. https://doi.org/10.1055/s-1983-30319.

(6) Pop, F.; Seifert, S.; Hankache, J.; Ding, J.; Hauser, A.; Avarvari, N. Modulation of the Charge Transfer and Photophysical Properties in Non-Fused Tetrathiafulvalene-Benzothiadiazole Derivatives. *Org. Biomol. Chem.* **2015**, *13* (4), 1040–1047. https://doi.org/10.1039/C4OB02100B.

(7) Rodríguez, J. G.; Martín-Villamil, R.; Felix, H. C.; Fonseca, I. Synthesis of 1,4-Di(n-Pyridyl)Buta-1,3-Diyne and Formation of Charge-Transfer Complexes. X-Ray Structure of 1,4-Di(3-Pyridyl)Buta-1,3-Diyne. *J.Chem.Soc., Perkin Trans.* **1997**, *1*, 709–714.

(8) Bartucci, M. A.; Ciszek, J. W. Substituent Parameters Impacting Isomer Composition and Optical Properties of Dihydroindolizine Molecular Switches. *J. Org. Chem.* **2014**, *79* (12), 5586–5594. https://doi.org/10.1021/jo500752p.

(9) Krause, L.; Herbst-Irmer, R.; Sheldrick, G. M.; Stalke, D. Comparison of Silver and Molybdenum Microfocus X-Ray Sources for Single-Crystal Structure Determination. *J. Appl. Crystallogr.* **2015**, *48* (1), 3–10. https://doi.org/10.1107/S1600576714022985.

(10) Sheldrick, G. M. SHELXT – Integrated Space-Group and Crystal-Structure Determination. *Acta Crystallogr. Sect. A Found. Adv.* **2015**, *71* (1), 3–8. https://doi.org/10.1107/S2053273314026370.

(11) Sheldrick, G. M. Crystal Structure Refinement with SHELXL. *Acta Crystallogr. Sect. C Struct. Chem.* **2015**, *71* (1), 3–8. https://doi.org/10.1107/S2053229614024218.
